# Pelvic floor complaint-related psychological distress recorded by pelvic physical therapists in the Netherlands: Additional analysis of data from an exploratory file review study

**DOI:** 10.12688/openreseurope.22137.1

**Published:** 2026-01-09

**Authors:** Alma Brand, Wim Waterink, Xynthia Kavelaars, Jacques van Lankveld

**Affiliations:** 1 Faculty of Psychology, Open University of The Netherlands, Heerlen, The Netherlands

**Keywords:** pelvic floor complaints, psychological distress, complaint patterns, latent class analysis

## Abstract

**Background:**

Patients with pelvic floor complaints (PFCs) experience various levels of PFC-related psychological distress along with their complaints. Paying attention to PFC-related psychological distress may enhance the intended biopsychosocial treatment approach in pelvic physical therapy practice. To our knowledge, the extent to which this distress is recorded by pelvic physical therapists in association with different combinations of PFCs has not been previously investigated.

**Aim:**

This exploratory retrospective file review study provides a first impression of the PFC-related distress recorded by pelvic physical therapists along with (combinations of) PFCs.

**Method:**

Pelvic physical therapists documented PFCs and PFC-related distress as recorded in patient files in an online survey. Descriptive statistics and correlations were calculated, and latent class analyses were performed to gain insight into profiles of PFCs and associated psychological distress.

**Results:**

A model with five profiles of PFCs and PFC-related psychological distress was selected. These profiles appear to be related to pregnancy and parity. A substantial proportion of distress was recorded with two profiles. One profile, characteristic of pregnant patients, indicated a high probability of defecation and micturition problems and pelvic pain, including a high likelihood of insecurity, loss of control, and disappointment. The other profile, characteristic of nulliparous patients, indicated a high probability of painful intercourse and micturition problems, including a high likelihood of disappointment, insecurity, helplessness, and sexual distress.

**Conclusion:**

The results show encouraging proof of attention given to PFC-related distress in pelvic physical therapy practice, despite this not being the primary focus of pelvic physical therapists. Based on our data, it remains unclear whether the psychological impact of PFCs receives sufficient attention in pelvic physical therapy. The results could also indicate the need for a more prominent position for PFC-related distress in patient files.

## Plain language summary

In pelvic physical therapy practice, therapists are expected to address their patients' physical, psychological, and social problems. Patients with pelvic floor complaints (PFCs) experience psychological distress along with their PFCs. It is unknown how the psychological distress that patients express is documented in patient files. Therefore, we conducted this file review study to obtain a first impression of the combination of PFCs and psychological distress recorded by pelvic physical therapists. The data were collected in an online survey. We analyzed frequencies, associations between PFCs and psychological distress, and complaint and distress profiles. Five clinically relevant profiles emerged that appear to be related to pregnancy and parity. With two profiles, a substantial proportion of distress was recorded. One profile, characteristic of pregnant patients, indicated a high probability of defecation and micturition problems and pelvic pain, along with a high likelihood of feelings of insecurity, loss of control, and disappointment. The other profile, characteristic of nulliparous patients, indicated a high probability of painful intercourse and micturition problems, along with a high likelihood of feelings of disappointment, insecurity, helplessness, and sexual distress. The results show encouraging proof that pelvic physical therapists pay attention to PFC-related psychological distress in clinical practice. However, it remains uncertain whether the psychological impact of PFCs receives sufficient attention in pelvic physical therapy. The results could also indicate that patient files lack adequate space to document PFC-related distress.

## Introduction

Following the Dutch pelvic physical therapy competency profile ^1^, pelvic physical therapists (PPTs) are trained to approach diagnosis and treatment of pelvic floor complaints (PFCs) from a biopsychosocial perspective ^2– 5^. With this holistic approach, it is acknowledged that PFCs are not solely biological or physical complaints but result from complex interactions among biological, psychological, and social factors, which should therefore all be considered equally for patients' well-being ^6^. PPTs should, thus, assess the presence and severity of their patients’ PFCs, the PFC-related restrictions, psychological distress, and social impact to help restore their patients’ daily, social, and sexual functioning to the best possible level ^1^. Omitting any of these biopsychosocial factors may jeopardize a holistic treatment approach and the intended duration and effectiveness of treatments provided.

In pelvic physical therapy education and practice, common PFCs such as urinary and fecal incontinence, micturition and defecation problems, pelvic organ prolapses, pelvic pain, and painful intercourse are often symptomatic of female pelvic floor dysfunction ^7^. Education and clinical practice are usually focused on the main PFC, rather than combinations of PFCs. Multiple PFCs may induce more distress than a single PFC and result from, or in, more complex psychosocial problems. In a recent Latent Class Analysis (LCA) study, the co-occurrence of common PFCs among women with specific parity status was examined. This analysis yielded five clinically relevant profiles of the PFCs. Two profiles were characteristic of pregnant patients, showing that in this patient group, among other PFCs, pelvic pain, defecation, and micturition problems were most likely to be encountered ^8^. Two profiles were characteristic of parous patients, showing that in this patient group, among other PFCs, pelvic organ prolapses, urinary incontinence, fecal incontinence, and defecation problems were most probable ^8^. One profile was characteristic of nulliparous patients, showing that in this patient group, among other PFCs, painful intercourse and micturition problems prevailed ^8^. These results, focusing solely on the presence of PFCs, suggest that examining PFC profiles may offer benefits for a holistic treatment approach and enhance treatment outcomes by focusing on the co-occurrence of PFCs, rather than the main PFC only ^8^.

Prior research also showed PFC-related distress to be a stronger predictor of receiving pelvic physical therapy treatment than PFC severity ^9^, highlighting the importance of accurately assessing and communicating psychological distress in the context of biopsychosocial history-taking, clinical reasoning, and treatment planning. A paucity of education regarding the communication of psychological distress in cases of severe PFCs might result in the omission, neglect, or failure to acknowledge existing distress. This, in turn, may have a deleterious effect on emotional and behavioral treatment outcomes, thereby rendering the treatment less effective because important predisposing or perpetuating factors for PFCs remain unaddressed or unresolved ^10^. To our knowledge, the effect of paying attention to PFC-related distress on treatment outcomes in pelvic physical therapy practice has not been investigated.

PFCs are also frequently accompanied by sexual problems and sexual distress ^11^. In clinical practice, open communication about sensitive topics such as sexual problems and psychological issues is often avoided due to discomfort discussing these topics, a lack of the necessary competencies and communication skills, and the fear of being negatively judged ^12^. Enhancing PPTs’ competencies in discussing these issues may facilitate a shift in discourse, reduce perceived taboos, encourage openness to sensitive topics, and increase understanding of patients’ problems. It should not be taken for granted that psychological distress will be resolved automatically by reducing the presence and severity of PFCs, as it has been shown that psychological distress can also be a predisposing factor to PFCs ^11, 13^. For example, when a female patient attends a PPT because of painful intercourse, she may feel that she is letting her partner down. The fact that she had a negative experience when her previous partner terminated their relationship due to this same problem causes high levels of psychological distress. She fears her new relationship may end in the same manner. Instructing and teaching this patient to relax her pelvic floor muscles without translating this ability toward the functional (private) context of relaxation during sexual intercourse, without addressing her psychological distress in interaction with her partner, may not solve her problem adequately. The patient’s distress may hinder her ability to relax her pelvic floor muscles during sexual intercourse, leaving her with unresolved painful intercourse.

Transparency about the nature and normalcy of sexual problems and PFC-related distress may further facilitate biopsychosocial treatment approaches, multidisciplinary collaboration with psychologists or sexologists, and benefit patients ^14^. Specific knowledge about predisposing psychological issues and an understanding of common types of PFC-related distress may lower barriers to discussing this in clinical practice. Therefore, it seems essential that PPTs relate to their patients by speaking the same language, considering word choice, interpretation, and the personal significance and importance of predisposing and PFC-related psychological distress in building mutual trust, facilitating shared decision-making, enhancing a sense of self-efficacy, and promoting better adherence to treatment ^15, 16^. Different types of distress may require different counseling or treatment approaches. This topic would receive more attention if it were given a more prominent place in education and in patient files. Not identifying, addressing, and interpreting psychological issues adequately could result in under-recording distress and in neglecting and underestimating the association between psychological distress and PFCs on patients’ lives and well-being, and ultimately, jeopardize treatment outcomes ^15, 16^. PPTs must keep their files up to date and meet the quality standards set by their professional society and insurance companies. Adapting patient files to reflect the biopsychosocial model equally may be a first step toward raising awareness of and improving documentation of psychological and social issues related to PFCs, thereby further enhancing the quality of their work.

Until recently, the literature gave a fragmented picture of PFC-related distress, primarily related to a specific PFC affecting patients’ daily, social, or sexual functioning. For example, in pelvic pain patients, anxiety (22.8–79.0%) and depression (14.0–56.9%) were found to be highly prevalent ^17^. Pelvic organ prolapses, incontinence, and painful intercourse are highly associated with body image issues, such as shame, embarrassment, and low self-esteem ^18– 23^. The latter observation stimulated a mixed-methods study into this topic ^24^. The results showed combinations of distinct types of distress being experienced by pregnant, parous, and nulliparous patients who do and do not receive pelvic physical therapy treatment. It also yielded a conceptual model that comprised seven distinct types of PFC-related distress, based on the expert opinions of medical and psychological pelvic healthcare providers, and patients with PFCs ^25^. The emergent types of PFC-related distress were: feeling insecure, feeling angry, feeling wronged, feeling helpless, feeling disappointed, loss of control, and sexual distress ^25^. The conceptual model demonstrated that insecurity is closely connected to the other six types of PFC-related distress, as this cluster occupies a central position in the model ^25^. It is unclear to what extent PPTs recognize and address these frequently co-occurring types of PFC-related distress in their history-taking and treatment approaches.

In pelvic physical therapy practice, questionnaires, including the Patient-specific Functional Scale ^26^, the Pelvic Floor Distress Inventory, the Pelvic Floor Impact Questionnaire (PFIQ) ^27^, and the Pelvic Organ Prolapse/Urinary Incontinence Sexual Questionnaire (PISQ) ^28, 29^, are frequently used to assess and evaluate patients’ PFCs and gain insight into the impact of PFCs and their accompanying distress. However, these questionnaires prioritize the restrictions ^26^, the frequency of PFC-related bother, and PFC-related restrictions affecting daily and sexual functioning ^27– 29^. They focus primarily on the somatic aspects of PFCs and lack specific questions about experienced psychological distress related to PFCs. One question in the PFIQ addresses distress, specifically asking about feelings of frustration ^27^. The PISQ addresses frequencies of experienced fear, disgust, embarrassment, and guilt when experiencing bother of PFCs during sexual activities ^28, 29^. As a result, the responses do not provide a comprehensive assessment of experienced PFC-related distress.

Because of the previous lack of clarity about PFC-related distress, it may have been challenging to train PPTs on this topic, providing them with tools and resources to capture their patients’ psychological distress ^1^. This may also explain why psychological distress and social well-being lack the prominent position that the somatic aspects of PFCs occupy in the formats of many pelvic physical therapy patient records. These factors result in training and facilitating PPTs who are more somatically focused and may lack adequate skills to address and communicate PFC-related distress and their patients' behavioral reactions ^1, 6, 10^, limiting the biopsychosocial character of their treatment.

Because PFCs were found to cluster by parity status, and because women with varying parity statuses experience different types of PFC-related distress, it is also possible that distress is part of these profiles ^9, 24, 25^. Combined profiles of PFCs and psychological distress (PFC+PD profiles) may assist PPTs in their biopsychosocial treatment approach by incorporating psychological factors into their history-taking, linking PFC-related distress to presented PFCs, facilitating clinical reasoning, informing treatment choices, and promoting multidisciplinary collaboration. PFC+PD profiles could facilitate communication about PFC-related distress in clinical practice. PFC+PD profiles may also facilitate patient comprehension of how the PFCs and PFC-related distress combine that they experience in conjunction with their pregnancy and parity status. This could help manage patients’ expectations when discussing treatment options and prognoses with PPTs ^8^.

Therefore, in this study, PFC+PD profiles were explored. We aimed to examine whether certain (combinations of) PFC-related distress are more common in specific PFC profiles. The purpose of collecting data on recorded PFC-related distress was novel and exploratory, acknowledging that unrecorded distress did not imply its absence. Therefore, this paper presents an exploratory secondary analysis of PPTs’ recordings of PFCs and PFC-related distress, building on the PFC profiles from the primary analysis. For this reason, the dataset used in the primary study was revisited for secondary analysis ^8^.

## Method

The present study employed the same research design, methodology, and data as the primary study on PFC profiles ^8^. In this method section, a concise description is provided to elucidate the procedures already undertaken, giving readers an understanding of the method employed in both the primary and current studies. The text was rewritten and condensed wherever possible to avoid the issue of self-plagiarism. However, overlap could not be avoided in some instances for clarity reasons.

### Ethical considerations

The Ethical Review Board of the Open University of the Netherlands approved the study protocol (May 29th, 2019/No. U2019/03973/HVM). Both PTs and patients provided their written informed consent before participation.

### Design

In this exploratory file review study, PPTs completed a brief eligibility questionnaire, which included questions regarding their qualifications, registration, employment status, work experience, and workplace settings. PPTs were asked to enter anonymized data from self-selected patient files in an online survey, after obtaining written permission from the included patients ^8^.

### Participants

**PPT inclusion and recruitment:** Registered, practicing Dutch PPTs were included in this study. Two Dutch pelvic physical therapy societies published calls to participate in their newsletters to help recruit PPTs. PPTs were also recruited verbally and online through LinkedIn and Facebook. PPTs were offered a reward for participation in the form of permanent education credits.

**Patient file selection:** Each PPT could include data from up to 30 patient files of pregnant, parous, and nulliparous patients aged between 18 and 45. These patients were either currently being treated or had received treatment within the past year. To explore the unbiased influence of pregnancy and parity, eligible pregnant patients were those expecting their first child; parous patients were those who had given birth no more than two years ago and were not pregnant; and nulliparous patients had no children and were not pregnant. The initial idea was to ask PPTs to include ten pregnant, ten parous, and ten nulliparous patients to explore profiles in equally large groups. However, this request was challenging due to the different caseloads and specializations. Therefore, it was decided that all submissions up to a maximum of 30 patients were deemed acceptable.

### Measurement instrument

PFCs and PFC-related distress recorded in patient files were to be entered in an online survey. Age, pregnancy status, gestational age in months, parity status, and the number of previous pregnancies were recorded for pregnant patients. The number of children, the age of the youngest child, and the number of vaginal deliveries and cesarian sections were inventoried for parous patients. A catalog was provided to assist in extracting the recorded PFCs and PFC-related distress from patient files before entering them in the online survey. PFC symptoms and definitions of the types of PFC-related distress from the conceptual model were included in the catalog (see Table 1).

**Table 1.** Reference catalog of pelvic floor symptoms and psychological distress definitions.

Catalog items		Symptoms and Definitions	NVFB Diagnoses	Categorized PFC
Pelvic Floor Complaints (PFC)				
**1**	**Urinary** **Incontinence**	Involuntary loss of urine when sneezing, coughing, laughing, jumping etc., Urge incontinence, Loss of urine during sex, Unnoticed loss of urine, or Leakage after micturition	Stress Urinary Incontinence (SUI) Urge Urinary Incontinence (UUI) Mixed Urinary Incontinence (MUI)	**Urinary** **Incontinence**
**2**	**Fecal Incontinence**	Involuntary loss of feces, Unnoticed loss of feces, Involuntary loss of feces when sneezing, coughing, laughing, jumping etc.	Fecal Incontinence Soiling Stamping	**Fecal** **Incontinence**
**3**	**Flatus**	Inability to hold wind, Excessive intestinal gas formation and bother of flatus, Flatus related to specific types of food	Anal Flatus	
**4**	**Dysfunctional** **Voiding**	Hesitation, Incomplete voiding, Pushing to urinate, Changed flow, Postponing micturition, Not taking time for micturition	Dysfunctional Voiding Urinary Retention	**Micturition** **Problems**
**5**	**Urge/Frequency** **(micturition)**	Frequent micturition, Frequent urge, Imperative urge, Painful urge, Interstitial Cystitis	Frequency Interstitial Cystitis Overactive Bladder	
**6**	**Urinary Tract ** **Infections**	Burning pain during micturition, Loss of control over urge, Sense of pressure in/on bladder, Frequent urge	Urinary Tract Infections	
**7**	**Urge/Frequency** **(defecation)**	Empty insistence, Incomplete emptying, Frequent urge, Difficulty postponing urge	Fecal Urge Frequency	**Defecation** **Problems**
**8**	**Constipation**	Having to push (hard) to empty bowel, Feeling bloated or full, Abdominal pain because of full bowel, No appetite, Postponing defecation, Overflow	Constipation Slow Transit Spastic Colon	
**9**	**Anal Complaints**	Hemorrhoids, Fissures, Pain during defecation, Anal cramp	Anal Cramp Anal Fissures	
**10**	**Vaginal Pelvic** **Organ Prolapse**	Sense of vaginal pressure, Heaviness, Sense of ball protruding, Visible ball protruding, Tampons do not stay put, Cystocele, Rectocele, Uterus prolapse	Cystocele Descensus Uteri Prolapse Rectocele	**Pelvic Organ** **Prolapse**
**11**	**Rectal Pelvic Organ** **Prolapse**	Sense of anal pressure, Protrusion of rectum through anus	Enterocele Prolapse	
**12**	**Low Back/Pelvic** **Pain**	Low back pain, Hernia, Pelvic pain, SI-joint pain, Buttock pain, Groin pain, Hip problems, Pain that spreads into the legs, Starting stiffness	Low Back Pain Pelvic Pain Pregnancy-related Pelvic Pain (pre- and postnatal) ^30^ Luxation	**Pelvic** **Pain**
**13**	**Genital Pain**	Perineal pain, Vulvodynia, Painful scars	Pelvic Floor Pain Scar Tissue Lacerations	
**14**	**Coccyx Pain**	Coccyx pain during sitting, sitting down and standing up from sitting	Coccygodynia	
**15**	**Painful Intercourse**	Penetration pain, Inability to use tampons, Inability to insert a finger in vagina, Vaginismus, Deep vaginal pain during penetration sex, Orgasm problems resulting from pelvic floor muscle tension	Dyspareunia Vaginismus Vulvar Pain Syndrome Vulvar Vestibulitis Syndrome	**Painful** **Intercourse**
Psychological Distress				
**19**	**Loss of Control**	Being unable to control one’s feelings or actions		
**20**	**Feeling Insecure**	Feeling uncertain or anxious about oneself		
**21**	**Feeling Wronged**	Being treated unfairly, or unjustly		
**22**	**Feeling Helpless**	The inability to defend oneself or act without help		
**23**	**Sexual Distress**	Distress related to problems in the sexual response cycle that prevents someone from experiencing satisfaction from sexual activities		
**24**	**Feeling Angry**	Feeling or showing strong annoyance, displeasure, or hostility		
**25**	**Feeling** **Disappointed**	Feeling sad or displeased because someone or something has failed to fulfil one’s hopes and expectations		

### Procedure

The file review study was embedded in the secure web-based application O4U ( https://o4u.ou.nl/en/node/234). The survey functionality in O4U was pilot-tested and adapted by the principal investigator, then retested and approved by two other PPTs. Participating PPTs received access to an information letter explaining the study’s background and objectives via an online link on the study platform. PPTs completed a short eligibility survey after signing the online informed consent form to gain access to the patient questionnaires. Download links were provided to access the informed consent forms for prospective patients. Printed informed consent forms were sent by regular mail on request. The patients signed informed consent forms that were posted to the principal investigator (AB) and stored in compliance with applicable privacy laws and regulations. Entering data online took a few minutes per patient. After a sensitivity analysis showed no relevant differences, the test data were added to the dataset to increase the sample size.

### Data-analysis

Data were analyzed using SPSS-28 ^31^, Excel, and R ^32^. Before the analysis, the entered PFCs were aligned with the categorization used in previous research, as indicated in Table 1 ^24^. The sum scores of each PFC were calculated based on the recorded number of symptoms shown in the catalog. Scores of ‘0’ indicated absence in patient files, and all other scores indicated presence, indicated by ‘1’, resulting in a data set of binary variables.

Descriptive statistics were calculated in SPSS and Excel, encompassing the recorded frequencies and percentages of the types of PFCs and PFC-related distress in the total sample and subgroups of pregnant, parous, and nulliparous patients. To account for their dichotomous measurement level, pairwise correlations between the proportions of the outcome variables were computed using the formula: r = (y(11) – y1*y2)/(y1*(1-y1)*y2*(1-y2))^(1/2) ^23^. In this formula, y(11) represents the proportion of patients with both complaints recorded, y1 is the proportion of patients for whom complaint ‘1’ was recorded, and y2 reflects the proportion of patients for whom complaint ‘2’ was recorded.

Latent Class Analysis (LCA) was performed to identify characteristic response patterns among the recorded PFCs and PFC-related distress and to cluster patients according to these patterns to identify typical patient profiles. The recommended sample size for a (multilevel) LCA is between 300 and 1000 respondents ^33^. In this LCA, PFC and psychological distress (PFC+PD) profiles were explored by adding the recordings of the seven types of PFC-related distress to those of the seven common PFCs. The LCA model selection process is explained in detail in the supplementary material (see Appendix B). Based on statistical considerations from log-likelihood indices and the Bayesian Information Criterion (BIC), the emergent models were explored to identify a preference for a single- or multilevel model (see Appendix B). The subsequent statistical model selection procedure was iterative ^34^, aiming to identify a plausible number of classes while exploring potential dependencies among patients clustered by group and by PPT practice. This exploration was necessary to assess possible differences between PPTs in PFCs and PFC-related distress recordings, to identify PFC+PD profiles, and to determine the likelihood of encountering these profiles among pregnant, parous, and nulliparous patients ^34^. The LCA model selection procedure was extended to include theoretical considerations and to consult four expert PPTs to interpret the emergent LCA models for clinical relevance.

## Results

Twenty-two PPTs participated in this study. On average, they reported 12.59 working years (SD = 6.48) in private practice. Three PPTs also worked in hospitals and healthcare centers. Data on 366 patients were included in the analyses. Given the specific patient populations and specialization of PPTs, the intended data stratification into three equally large groups of pregnant, parous, and nulliparous patients failed. Data from 76 pregnant patients with a mean age of 30.11 years (SD = 3.68) and gestation age of 23.59 weeks (SD = 7.64), 205 parous patients with a mean age of 32.83 years (SD = 4.11), and on average 1.61 children, and 85 nulliparous patients with a mean age of 28.69 years (SD = 8.17) were analyzed.

Insecurity was the most frequently recorded type of PFC-related distress. Loss of control and sexual distress followed as the second and third, before disappointment, helplessness, anger, and finally feeling wronged. The highest percentage of loss of control was recorded for patients with fecal incontinence. The highest percentages of sexual distress and disappointment were recorded for patients experiencing painful intercourse. Table 2 shows the percentages of recorded types of PFC-related distress for the total sample and each specific PFC.

**Table 2.** Distribution of the types of pelvic floor complaint-related distress in the full sample and across pelvic floor complaints.

		Types of Pelvic Floor Complaint-related Distress						
Complaints	Sample size	Loss of Control	Feeling Insecure	Feeling Wronged	Feeling Helpless	Sexual Distress	Feeling Angry	Feeling Disappointed
	N	%	%	%	%	%	%	%
**Full Sample**	**366**	14.6	**27.4**	1.6	10.0	14.1	3.0	12.5
Urinary Incontinence: **UI**	**125**	20.0	**33.6**	2.4	9.6	14.4	4.0	13.6
Fecal Incontinence: **FI**	**20**	**30.0**	**35.0**	0.0	15.0	5.0	5.0	5.0
Micturition Problems: **MP**	**131**	18.3	**30.5**	0.0	13.7	18.3	3.1	15.3
Defecation Problems: **DP**	**113**	15.9	**29.2**	0.0	10.6	18.6	3.5	14.2
Pelvic Organ Prolapse: **POP**	**72**	15.3	**25.0**	1.4	6.9	9.7	2.8	11.1
Pelvic Pain: **PP**	**267**	16.1	**27.3**	2.2	12.0	13.1	3.7	13.5
Painful Intercourse: **PI**	**86**	20.9	**45.3**	0.0	16.3	**34.9**	5.8	**27.9**

Note: The types of PFC-related distress with recordings of more than 25% in the full sample and per pelvic floor complaint are highlighted in bold.

Table 3 shows the pairwise correlations between PFCs and types of PFC-related distress. Positive correlations indicate complaints likely to be (not) recorded together, and negative correlations indicate that one complaint is likely to be recorded but not the other. The correlation between painful intercourse and sexual distress was moderate to strong. A weak to moderate correlation was found between painful intercourse and insecurity and disappointment, and between micturition problems and loss of control. Weak positive and negative correlations were found between various PFCs and types of PFC-related distress. Urinary incontinence and feeling wronged were least likely to be recorded together.

**Table 3.** Pairwise correlations in the full sample between pelvic floor complaints and pelvic floor complaint-related distress.

		Types of Pelvic Floor Complaint-related Distress					
Complaints	Loss of Control	Feeling Insecure	Feeling Wronged	Feeling Helpless	Sexual Distress	Feeling Angry	Feeling Disappointed
Urinary Incontinence: **UI**	**0.10**	**0.08**	**-0.20**	-0.01	**0.00**	**0.04**	**0.02**
Fecal Incontinence: **FI**	**0.10**	**0.04**	-0.03	**0.04**	-0.06	**0.03**	-0.05
Micturition Problems: **MP**	**0.20**	**0.04**	-0.10	**0.08**	**0.08**	**0.00**	**0.06**
Defecation Problems: **DP**	**0.02**	**0.02**	-0.09	**0.01**	**0.08**	**0.02**	**0.03**
Pelvic Organ Prolapse: **POP**	**0.01**	-0.03	-0.01	-0.05	-0.06	-0.01	-0.02
Pelvic Pain: **PP**	**0.05**	-0.01	**0.08**	**0.08**	-0.04	**0.07**	**0.04**
Painful Intercourse: **PI**	**0.09**	**0.19**	-0.07	**0.11**	**0.42**	**0.09**	**0.24**

Note: **N = 366.** In bold, positive correlations are highlighted. In green, positive correlations above 0.10 are indicated. In red, negative correlations below 0.10 are indicated.

### Latent Class Analysis

The model selection procedure indicated that the data best supported a 5-class multilevel group LCA. This model had pregnancy and parity status as groups, and PPT as a cluster variable (see Appendix B for more details on model selection). Model selection was based on both statistical indicators (log-likelihood, BIC) and theoretical considerations. In the theoretical considerations, the plausibility of PFC combinations was given more weight than that of PFC-related distress combinations, because the somatic PFC recordings were assumed to be more robust and structured than the PFC-related distress recordings.

**Figure 1.**
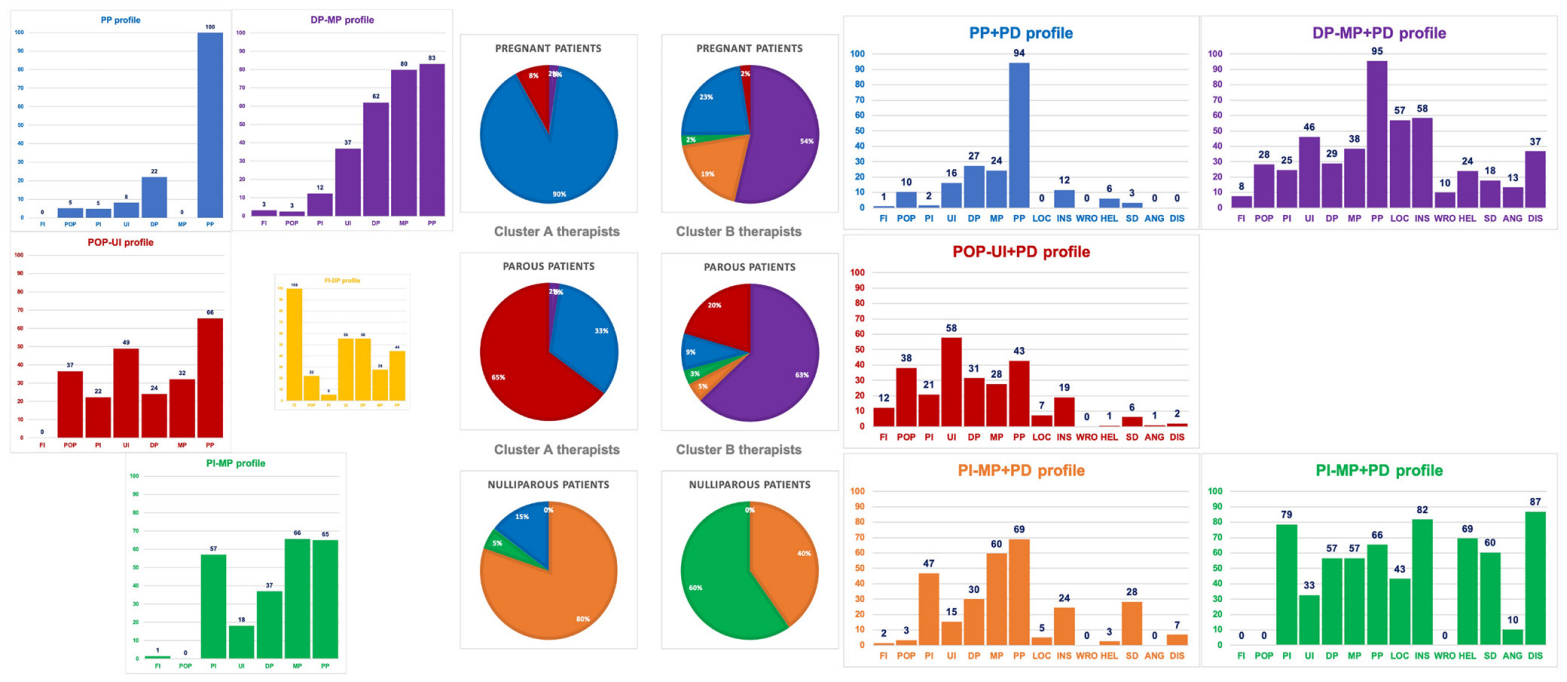
Five relevant pelvic floor complaint and pelvic floor complaint-related distress profiles, and the likelihood of each profile in each patient group, as recorded by pelvic physical therapists. Note: FI = Fecal incontinence, POP = Pelvic organ prolapse, PI = Painful intercourse, UI = Urinary incontinence, DP = Defecation problems, MP = Micturition problems, PP = Pelvic pain. PFC = Pelvic floor complaint, PD = Psychological distress. The bar charts show the likelihood, in %, that each complaint is present in a profile. On the left, the original PFC-only profiles are shown for comparison with the combined PFC+PD profiles on the right. In the middle, the likelihood of each profile's presence in pregnant, parous, and nulliparous patient files is shown for the two groups of pelvic physical therapists. Cluster A therapists recorded very little PFC-related distress, as indicated in the pie charts on the far left, whereas Cluster B therapists recorded more PFC-related distress.

Similar to the earlier work, the five classes (profiles) found were named after the one or two most distinct pelvic floor complaints with the highest likelihood ^8^. As the somatic component of the profiles showed a strong correspondence with the earlier work, the same names and colors were used to facilitate interpretation and comparison between profiles with and without indicators of psychological burden. In Figure 1, the PFC+PD profiles are shown alongside the PFC-only profiles from our earlier work ^8^.

Two clusters of therapists were identified, and the clustering appeared to be related to differences in the overall frequency of recorded distress among PPTs. The class probabilities per therapist cluster are also visualized in Figure 1, split by parity status. The left column of three pie charts in the center of Figure 1 shows the distribution of PFC+PD profiles across patient groups among Cluster A PPTs, who were generally found to record psychological distress less often. The right column of the three pie charts shows the distribution of PFC+PD profiles across patient groups among Cluster B PPTs, who were generally found to record psychological distress more often.

The five PFC+PD profiles found were: 1) pelvic pain (blue); 2) defecation and micturition problems (purple); 3) pelvic organ prolapse and urinary incontinence (red); 4) painful intercourse and micturition problems (green); and 5) painful intercourse and micturition problems (orange). In the pelvic pain PFC+PD profile (blue), the likelihood of pelvic pain was high, with low probabilities of the other PFCs. No substantial distress, other than insecurity, was recorded in this profile. Cluster A PPTs most frequently recorded this profile for pregnant patients, and with a lower probability for parous and nulliparous patients. The defecation and micturition problems PFC+PD profile (purple) also included high probabilities of pelvic pain, with moderate likelihood of defecation and micturition problems, pelvic organ prolapse, painful intercourse, and urinary incontinence, but not fecal incontinence. All types of distress were substantially recorded with this profile, in particular loss of control, insecurity, and disappointment. Cluster B PPTs most frequently recorded this profile in pregnant and parous patients, suggesting that this PFC+PD profile may not be exclusive to pregnant patients. The pelvic organ prolapse and urinary incontinence PFC+PD profile (red) was most characteristic of parous patients. No substantial psychological distress was recorded with this profile apart from insecurity. Cluster A PPTs recorded this profile most frequently. Next to the painful intercourse and micturition problems PFC+PD profile (green), a similar PFC+PD profile (orange) emerged. Both these green and orange painful intercourse and micturition profiles were most frequently recorded for nulliparous patients. One painful intercourse and micturition problems PFC+PD profile (green) showed high probabilities in psychological distress, particularly loss of control, insecurity, helplessness, sexual distress, and disappointment. The other painful intercourse and micturition problems PFC+PD profile (orange) included lower probabilities for various similar PFCs, and lower probabilities in psychological distress, including mainly insecurity and sexual distress. For both painful intercourse and micturition problems PFC+PD profiles, cluster A and B PPTs recorded similar types of distress at different frequencies, suggesting that cluster B PPTs were more likely to record distress than cluster A PPTs. The results were discussed with four PPT experts, who identified the five PFC+PD profiles as clinically relevant.

### Comparison between the PFC-only and PFC+PD profiles

The pelvic pain (blue) and defecation and micturition problems (purple) profiles remained most characteristic of pregnant patients. The two pelvic pain profiles (blue) showed high probabilities of pelvic pain and low probabilities of the other PFCs. Both defecation and micturition problem profiles (purple) included high probabilities of pelvic pain, and after adding psychological distress, the likelihood of defecation and micturition problems was lower. In both pelvic organ prolapse and urinary incontinence profiles (red), the probability of the various types of PFCs and their diffuse character was similar. The fecal incontinence and defecation problems profile (yellow) disappeared completely after psychological distress was added to the analyses. This could be due to the relatively small group of twenty parous patients for whom fecal incontinence was recorded. In contrast, cluster B therapists recorded the micturition and defecation PFC+PD profile (purple), which includes a higher probability of defecation problems, also in files from parous patients. In a new study with a larger sample, the fecal incontinence and defecation problems profile (yellow) might re-emerge. In this multilevel LCA, the fecal incontinence recordings appear to have been merged into the pelvic organ prolapse and urinary incontinence PFC+PD profile (red), confirming these PFCs as characteristic of parous patients. The defecation problem recordings may have been incorporated into the micturition and defecation problems profile (purple), which, in this study, was also frequently recorded by cluster B therapists for parous patients. The multilevel painful intercourse and micturition problems PFC+PD profile (green) showed similar high probabilities in PFCs to the PFC-only profile (green).

## Discussion

Based on data from patient files, frequencies and associations between PFCs and types of PFC-related distress were explored to identify clinically relevant PFC+PD profiles. Five statistically sound and clinically relevant PFC+PD profiles related to pregnancy and parity were found. Two PFC+PD profiles were characteristic of pregnant patients, one of parous patients, and two of nulliparous patients. While some attention is given to PFC-related distress, it remains underrepresented in the patient files. In the profiles, sparse recordings of PFC-related distress appeared to be associated with PPT characteristics and the nature, number, and combinations of probable PFCs in each profile.

PPTs mostly recorded three types of PFC-related distress from the conceptual model: ‘feeling insecure’, ‘loss of control’, and ‘sexual distress’ with different PFC profiles ^25^. The other kinds of PFC-related distress -‘feeling helpless’, ‘feeling wronged’, ‘feeling angry’, and ‘feeling disappointed’- were less frequently recorded. This observation raises questions about the transparency and accuracy of PPTs’ communication with their patients regarding the interpretation and documentation of expressed PFC-related distress ^16^.

The high frequency with which insecurity was recorded in comparison to other types of distress suggests that PPTs may easily label any distress expressed by patients as insecurity. Although it may seem positive that insecurity is recorded, it remains uncertain whether it captures the full range of distress experienced by patients. The knowledge that insecurity is related to other forms of distress ^25^ warrants further exploration of the factors contributing to the observed insecurity, as illustrated in the following examples. Losing control over one’s body may trigger a sense of insecurity due to the inability to perform daily activities. Sexual function problems may trigger insecurity for not living up to one’s own and one’s partner’s expectations during sexual interactions. Anger may trigger insecurity, leading to doubts about one’s ability to recover. Helplessness may trigger insecurity due to the loss of independence and autonomy. A sense of being wronged may trigger insecurity due to the belief that the odds are against you. Disappointment may trigger insecurity due to unfulfilled hopes and expectations ^24^. Further exploration of the interrelated types of distress to insecurity can enhance understanding of patients’ guiding questions and contribute to the development of more tailored treatment plans.

Loss of control may be more easily recognized because this type of distress has both physical and psychological characteristics. The physical inability to perform certain physical activities often equates to a loss of control over muscle function, resulting in restrictions or the inability to engage in daily, social, and sexual activities, which can lead to distress from losing control over one’s life. Given PPT’s inventory of functional restrictions, loss of control may be acknowledged sooner than other types of PFC-related distress because of this physical aspect. Therefore, it is uncertain to what extent PPTs acknowledge the psychological side of this type of distress.

Sexual distress may be more easily recognized in the specific and sensitive context of sexual function problems, which may explain the substantially higher frequency with which sexual distress is recorded. The sensitivity of discussing sexual function problems may contribute to heightened attention to distress, given that it is often distressing to discuss the topic of sexual function for both patients and healthcare providers ^35^. In addition to this observation, the dyadic nature of sexual function problems in the context of relationships, including partner involvement and behavior, and the wish for children, might also highlight this type of distress from a social perspective.

No literature was found on research that unravels PFC + PD profiles; therefore, it is not possible to compare the results with previous findings. However, the preliminary results in this study suggest distinct sets of PFC-related distress types for each profile. The results show some attention to PFC-related distress in pelvic physical therapy practice in line with the biopsychosocial perspective. More distress was recorded in two: the defecation and micturition problems (purple) and the painful intercourse and micturition problems (green) PFC+PD profiles with a higher probability of multiple PFCs than in the other: the pelvic pain (blue), the pelvic organ prolapse and urinary incontinence (red), and the painful intercourse and micturition problems (orange) PFC+PD profiles. A higher likelihood of multiple PFCs being presented together might automatically trigger a greater alertness to psychological distress, which may render the recording of distress more likely. In the blue, red, and orange profiles, one for each pregnancy and parity group, PFC-related distress was sparsely recorded, despite the high likelihood of one or more distinct PFC types. These findings do not appear to fully reflect the different kinds of PFC-related distress of pregnant, parous, and nulliparous patients, and the higher levels of PFC-related distress associated with, and predictive of, receiving pelvic physical therapy treatment as identified in prior research ^9, 24, 25^. Women receiving pelvic physical therapy treatment, particularly parous patients, expressed high levels of anger and feeling wronged, which does not show in the pelvic organ prolapse and urinary incontinence PFC+PD profile (red). Nulliparous patients expressed high levels of helplessness, which is only recorded in the painful intercourse and micturition problems PFC+PD profile (green). Pregnant patients expressed high levels of loss of control, which is only recorded in the defecation and micturition problems PFC+PD profile (purple) ^24^. The low likelihood of PFC-related distress recorded in parous patients' profiles, compared to the other profiles, raised the question of whether the distress experienced by parous patients was not evident enough or perhaps even taken for granted and therefore not recorded. This stands in high contrast with the recent evidence of distinct and overwhelming types of distress being experienced by parous patients who receive pelvic physical therapy treatment ^24, 25^. A possible explanation is that the treatment of parous patients primarily focuses on the recovery of pelvic floor muscle function after varying degrees of pelvic floor damage, including stretching, lacerations, and episiotomies, which may prompt more somatically-oriented guiding questions and treatment approaches. This observation warrants further exploration of the reasons and opportunities for recording or not recording psychological distress in pelvic physical therapy practice.

When PFC-related distress was not recorded, this did not necessarily imply its absence, as patients may not have expressed, discussed, or acknowledged it with PPTs, and it may not have been recorded for various reasons. The overall sparseness of PFC-related distress recordings may be due to various factors, such as a lack of recording space for psychological issues in patient files or limited attention to psychological problems and PFC-related distress in pelvic physical therapy education. Arguments for or against recording psychological distress in patient files often revolve around the balance between legal and ethical obligations, quality of care, client rights, and privacy ^1, 36^. Not recording distress may be related to the subject's sensitivity, a lack of patients’ permission to record their distress out of fear of stigma and taboo, or fear of breaches of privacy and confidentiality that accompany discussing these topics. Finally, the PPT’s subjective judgment regarding the relevance of distress to treatment and prognosis is decisive when it comes to recording psychological distress in patient files. Therefore, the profiles provide only a first impression of the types of PFC-related distress associated with each profile. It is also uncertain whether the conspicuous absence of psychological distress in the definition of pelvic floor dysfunction in comparison to the inclusion of distress in the definition of sexual dysfunction relates to the low recordings of PFC-related distress in patient files ^7, 37^. The sparse PFC-related distress recordings and expressed concerns regarding inadequate communication of patients' pain and behavioral responses may indicate a necessity to re-evaluate the application of the biopsychosocial framework within pelvic physical therapy education and practice ^8, 10^. It may be necessary to educate PPTs on how to better recognize various types of psychological issues and PFC-related distress and how to communicate them. In future research, patient reports should be included in analyses to verify the accuracy of PPT-recorded PFC-related distress. In addition to the presence of PFC-related distress, the severity, duration, and impact of distress should be considered to enhance the clinical utility of the combined profiles and improve the biopsychosocial treatment approach in pelvic physical therapy practice.

### Strengths and limitations

A file review design was chosen because it provides insight into and enhances understanding of healthcare processes, such as adherence to guidelines and protocols, the completeness and accuracy of clinical documentation, and opportunities for giving feedback and improving education. In the exploratory phase of research, a file review study is a practical and efficient approach. File review studies are designed to assess and improve the quality of care ^38^. For this reason, in this study, the attention to psychological distress in pelvic therapy practice was explored, given the intended biopsychosocial treatment approach, and our interest in the documentation of psychological distress in patient files. A file review study respects the privacy and anonymity of patients, including those who may not have initially wanted to participate, and avoids recall bias. File review studies are conducted to identify patterns in patient data, informing critical healthcare decisions, such as patient treatment outcomes and multidisciplinary collaboration. File review studies are valuable in situations where clinical details relevant to treatments may be easily overlooked or deemed irrelevant, and when the goal is to gain an overview of treatment content. Given the exploratory nature of this research, the retrospective study design was considered the most effective and suitable at this stage. Data were collected in alignment with the study's aim.

A limitation of this research is that the results cannot be generalized due to the stratified, non-representative sample and potential selection bias, as PPTs selected their files. In addition, PFC-related distress was sparsely recorded—for unknown reasons—and may have been incompletely, inconsistently, or not concisely documented, despite the given instructions, potentially resulting in information bias. Including patient-reported PFC-related distress should be considered in follow-up research to interpret the recorded PFC-related distress with more certainty.

## Conclusion

This exploratory retrospective file review study provides initial insight into how Dutch PPTs recorded PFC-related distress alongside PFCs in their patient files. While some PFC-related distress was recorded, especially insecurity, loss of control, and sexual distress, the overall recording was limited. Enhanced attention to predisposing psychological issues to PFCs and PFC-related distress in education and documentation could further support PPTs’ biopsychosocial approach in history-taking, (multidisciplinary) treatment selection and approaches, collaboration with psychologists or sexologists, and enhance patient care. In forthcoming research, further exploration of PFC+PD profiles from both patient and PPT perspectives is recommended to solidify the profiles. Adding information about severity, duration, and functional restrictions may further increase the clinical utility of the profiles.

## Data Availability

OSF: Pelvic Health Problems in Young Adult Women, Doi: https://doi.org/10.17605/OSF.IO/ZG2BN ^39^ This project contains the following underlying data: Data en Codeboek PPT studie-DEF-DEF.xlsx Function_bootstrap.R Functions_plot.R LCA.R Data are available under the terms of the Creative Commons Zero “No rights reserved” data waiver (CC0 1.0 Public domain dedication) ( http://creativecommons.org/publicdomain/zero/1.0/). OSF: Pelvic Health Problems in Young Adult Women, doi https://doi.org/10.17605/OSF.IO/DWNYH ^40^ This project contains the following data: Appendix A Appendix B.pdf Survey PPT Study.docx Data en Codeboek PPT studie-DEF-DEF.xlsx Data en Codeboek PPT studie-DEF-DEF NL&EN.xlsx Function_bootstrap.R Functions_plot.R LCA.R Data are available under the terms of the Creative Commons Zero “No rights reserved” data waiver (CC0 1.0 Public domain dedication) ( http://creativecommons.org/publicdomain/zero/1.0/).
