# Pelvic floor complaint-related psychological distress recorded by pelvic physical therapists in the Netherlands: Additional analysis of data from an exploratory file review study

**DOI:** 10.12688/openreseurope.22137.3

**Published:** 2026-10-10

**Authors:** Alma M. Brand, Wim Waterink, Xynthia Kavelaars, Jacques van Lankveld

**Affiliations:** 1 Faculty of Psychology, Open University of The Netherlands, Heerlen, The Netherlands

**Keywords:** pelvic floor complaints, psychological distress, complaint patterns, latent class analysis

## Abstract

**Background:**

Patients with pelvic floor complaints (PFCs) experience various levels of PFC-related psychological distress along with their complaints. Paying attention to PFC-related psychological distress may enhance the intended biopsychosocial treatment approach in pelvic physical therapy practice. To our knowledge, the extent to which this distress is recorded by pelvic physical therapists in association with different combinations of PFCs has not been previously investigated.

**Aim:**

This exploratory, hypothesis-generating retrospective file review study provides a first impression of the PFC-related distress recorded by pelvic physical therapists along with (combinations of ) PFCs.

**Method:**

Pelvic physical therapists documented PFCs and PFC-related distress as recorded in patient files in an online survey. Descriptive statistics and correlations were calculated, and latent class analyses were performed to gain insight into indicative profiles of PFCs and associated psychological distress.

**Results:**

A model with five profiles of PFCs and PFC-related psychological distress was selected. These profiles appear to be related to pregnancy and parity. A substantial proportion of distress was recorded with two profiles. One profile, most characteristic of pregnant patients, indicated a high probability of defecation and micturition problems and pelvic pain, including a high likelihood of insecurity, loss of control, and disappointment. The other profile, most characteristic of nulliparous patients, indicated a high probability of painful intercourse and micturition problems, including a high likelihood of disappointment, insecurity, helplessness, and sexual distress.

**Conclusion:**

The results show encouraging proof of attention given to PFC-related distress in pelvic physical therapy practice, despite this not being the primary focus of pelvic physical therapists. Based on our data, it remains unclear whether the psychological impact of PFCs receives sufficient attention in pelvic physical therapy. The results could also indicate the need for a more prominent position for PFC-related distress in patient files.

## Plain language summary

In pelvic physical therapy practice, therapists are expected to address their patients’ physical, psychological, and social problems. Patients with pelvic floor complaints (PFCs) experience psychological distress along with their PFCs. It is unknown how the psychological distress that patients express is documented in patient files. Therefore, we conducted this file review study to obtain a first impression of the combination of PFCs and psychological distress recorded by pelvic physical therapists. The data were collected in an online survey. We analyzed frequencies, associations between PFCs and psychological distress, and complaint and distress profiles. Five clinically relevant profiles emerged that appear to be related to pregnancy and parity. With two profiles, a substantial proportion of distress was recorded. One profile, characteristic of pregnant patients, indicated a high probability of defecation and micturition problems and pelvic pain, along with a high likelihood of feelings of insecurity, loss of control, and disappointment. The other profile, characteristic of nulliparous patients, indicated a high probability of painful intercourse and micturition problems, along with a high likelihood of feelings of disappointment, insecurity, helplessness, and sexual distress. The results show encouraging proof that pelvic physical therapists pay attention to PFC-related psychological distress in clinical practice. However, it remains uncertain whether the psychological impact of PFCs receives sufficient attention in pelvic physical therapy. The results could also indicate that patient files lack adequate space to document PFC-related distress.

## Introduction

Following the Dutch pelvic physical therapy competency profile, ^1^ pelvic physical therapists (PPTs) are trained to approach diagnosis and treatment of pelvic floor complaints (PFCs) from a biopsychosocial perspective. ^2^^–^^5^ With this holistic approach, it is acknowledged that PFCs are not solely biological or physical complaints but result from complex interactions among biological, psychological, and social factors, and that these factors should therefore all be considered equally for patients’ well-being. ^6^ This is especially important given the bidirectional relationship between PFCs and psychological distress, and the potentially negative influence of psychological distress on treatment results. ^7^^,^^8^ PPTs should thus assess the presence and severity of their patients’ PFCs, PFC-related restrictions, psychological distress, and the social impact of experiencing PFCs to help restore their patients’ daily, social, and sexual functioning to the best possible level. ^1^ Omitting any of these biopsychosocial factors may jeopardize a holistic treatment approach and the intended duration and effectiveness of treatments provided.

In pelvic physical therapy education and practice, common PFCs such as urinary and fecal incontinence, micturition and defecation problems, pelvic organ prolapses, pelvic pain, and painful intercourse are often symptomatic of female pelvic floor dysfunction. ^9^ Education and clinical practice are usually focused on the main PFC, rather than combinations of PFCs. Multiple PFCs may induce more distress than a single PFC and result from, or in, more complex psychosocial problems. In a recent Latent Class Analysis (LCA) study, the co-occurrence of common PFCs among women with specific parity status was examined. This analysis identified five indicative, clinically relevant PFC profiles. Two profiles appeared most characteristic of pregnant patients, suggesting that, in this patient group, among other PFCs, pelvic pain, defecation, and micturition problems were most likely to be encountered. ^10^ Two profiles appeared most characteristic of parous patients, suggesting that, in this patient group, among other PFCs, pelvic organ prolapses, urinary incontinence, fecal incontinence, and defecation problems were most probable. ^10^ One profile appeared most characteristic of nulliparous patients, suggesting that, in this patient group, among other PFCs, painful intercourse and micturition problems prevailed. ^10^ These results, which focus solely on the presence of PFCs, suggest that examining PFC profiles may offer benefits for a holistic treatment approach and enhance treatment outcomes by emphasizing the co-occurrence of PFCs rather than focusing on the main PFC only. ^10^

Prior research also showed PFC-related distress to be a stronger predictor of receiving pelvic physical therapy treatment than PFC severity, ^11^ highlighting the importance of accurately assessing and communicating psychological distress in the context of biopsychosocial history-taking, clinical reasoning, and treatment planning. A paucity of education regarding the communication of psychological distress in cases of severe PFCs might result in the omission, neglect, or failure to acknowledge existing distress. This, in turn, may have a deleterious effect on emotional and behavioral treatment outcomes, thereby rendering the treatment less effective because important psychological predisposing or perpetuating factors for PFCs remain unaddressed or unresolved. ^12^ To our knowledge, the effect of paying attention to PFC-related distress on treatment outcomes in pelvic physical therapy practice has not been investigated.

PFCs are also frequently accompanied by sexual problems and sexual distress. ^13^ In clinical practice, open communication about sensitive topics such as sexual problems and psychological issues is often avoided due to discomfort discussing these topics, a lack of the necessary competencies and communication skills, and the fear of being negatively judged. ^14^ Enhancing PPTs’ competencies in discussing these issues may facilitate a shift in discourse, reduce perceived taboos, encourage openness to sensitive topics, and increase understanding of patients’ problems. It should not be taken for granted that psychological distress will be resolved automatically by reducing the presence and severity of PFCs, as it has been shown that psychological distress can also be a predisposing factor to PFCs. ^13^^,^^15^ For example, when a female patient attends a PPT because of painful intercourse, she may feel that she is letting her partner down. The fact that she had a negative experience when her previous partner terminated their relationship due to this same problem causes high levels of psychological distress. She fears her new relationship may end in the same manner. Instructing and teaching this patient to relax her pelvic floor muscles without translating this ability toward the functional (private) context of relaxation during sexual intercourse, without addressing her psychological distress in interaction with her partner, may not solve her problem adequately. The patient’s distress may hinder her ability to relax her pelvic floor muscles during sexual intercourse, leaving her with unresolved painful intercourse.

Transparency about the nature and normalcy of sexual problems and PFC-related distress may further facilitate biopsychosocial treatment approaches, multidisciplinary collaboration with psychologists or sexologists, and benefit patients. ^16^ Specific knowledge about predisposing psychological issues and an understanding of common types of PFC-related distress may lower barriers to discussing this in clinical practice. Therefore, it seems essential that PPTs relate to their patients by speaking the same language, considering word choice, interpretation, and the personal significance and importance of predisposing and PFC-related psychological distress in building mutual trust, facilitating shared decision-making, enhancing a sense of self-efficacy, and promoting better adherence to treatment. ^17^^,^^18^ Different types of distress may require different counseling or treatment approaches. This topic would receive more attention if it were given a more prominent place in education and in patient files. Not identifying, addressing, and interpreting psychological issues adequately could result in under-recording distress and in neglecting and underestimating the association between psychological distress and PFCs on patients’ lives and well-being, and ultimately, jeopardize treatment outcomes. ^17^^,^^18^ PPTs must keep their patient files up to date and meet the quality standards set by their professional society and insurance companies. Adapting patient files to better reflect the biopsychosocial model may be a first step toward raising awareness of and improving documentation of psychological and social issues related to PFCs, thereby further enhancing the quality of their work.

Until recently, the literature gave a fragmented picture of PFC-related distress, primarily related to a specific PFC affecting patients’ daily, social, or sexual functioning. For example, in pelvic pain patients, anxiety (22.8–79.0%) and depression (14.0–56.9%) were found to be highly prevalent. ^19^ Pelvic organ prolapses, incontinence, and painful intercourse are highly associated with body image issues, such as shame, embarrassment, and low self-esteem. ^20^^–^^25^ The latter observation stimulated a mixed-methods study on this topic. ^26^ The results showed combinations of distinct types of distress being experienced by pregnant, parous, and nulliparous patients who do and do not receive pelvic physical therapy treatment. It also yielded a conceptual model that comprised seven distinct types of PFC-related distress, based on the expert opinions of medical and psychological pelvic healthcare providers, and patients with PFCs. ^25^ The emergent types of PFC-related distress were feeling insecure, feeling angry, feeling wronged, feeling helpless, feeling disappointed, loss of control, and sexual distress. ^27^ The conceptual model demonstrated that insecurity is closely connected to the other six types of PFC-related distress, as this cluster occupies a central position in the model. ^27^ It is unclear to what extent PPTs recognize and address these frequently co-occurring types of PFC-related distress in their history-taking and treatment approaches.

In pelvic physical therapy practice, questionnaires, including the Patient-specific Functional Scale, ^28^ the Pelvic Floor Distress Inventory, the Pelvic Floor Impact Questionnaire (PFIQ), ^29^ and the Pelvic Organ Prolapse/Urinary Incontinence Sexual Questionnaire (PISQ), ^30^^,^^31^ are frequently used to assess and evaluate patients’ PFCs and gain insight into the impact of PFCs and their accompanying distress. However, these questionnaires prioritize the restrictions, ^28^ the frequency of PFC-related bother, and PFC-related restrictions affecting daily and sexual functioning. ^29^^–^^31^ They focus primarily on the somatic aspects of PFCs and lack specific questions about experienced psychological distress related to PFCs. One question in the PFIQ addresses distress, specifically asking about feelings of frustration. ^29^ The PISQ addresses frequencies of experienced fear, disgust, embarrassment, and guilt when experiencing bother of PFCs during sexual activities. ^30^^,^^31^ As a result, the responses do not provide a comprehensive assessment of experienced PFC-related distress.

Because of the previous lack of clarity about PFC-related distress, it may have been challenging to train PPTs on this topic, providing them with tools and resources to capture their patients’ psychological distress. ^1^ This may also explain why psychological distress and social well-being lack the prominent position that the somatic aspects of PFCs occupy in the formats of many pelvic physical therapy patient records. These factors result in training and facilitating PPTs who are more somatically focused and may lack adequate skills to address and communicate PFC-related distress and their patients’ behavioral reactions, ^1^^,^^6^^,^^12^ limiting the biopsychosocial character of their treatment.

Because PFCs were found to cluster by parity status, and because women with varying parity statuses experience different types of PFC-related distress, it may also be possible that distress is part of these profiles. ^11^^,^^26^^,^^27^ Combined profiles of PFCs and psychological distress (PFC + PD profiles) may assist PPTs in their biopsychosocial treatment approach by incorporating psychological factors into their history-taking, linking PFC-related distress to the PFCs presented, facilitating clinical reasoning, informing treatment choices, and promoting multidisciplinary collaboration. PFC + PD profiles could facilitate communication about PFC-related distress in clinical practice. PFC + PD profiles may also facilitate patient comprehension of how the PFCs and PFC-related distress combine that they experience in conjunction with their pregnancy and parity status. This could help manage patients’ expectations when discussing treatment options and prognoses with PPTs. ^10^

Therefore, in this study, PFC + PD profiles were explored to generate hypotheses about characteristic combinations of PFCs and psychological distress for clinical practice. We aimed to examine whether certain (combinations of ) PFC-related distress are more common in specific PFC profiles. The purpose of collecting data on recorded PFC-related distress was novel and exploratory, acknowledging that unrecorded distress did not imply its absence. Therefore, this paper presents an exploratory and hypothesis-generating secondary analysis of PPTs’ recordings of PFCs and PFC-related distress, building on the indicative PFC profiles from the primary analysis. For this reason, the dataset used in the primary study was revisited for a secondary analysis. ^10^

## Method

The present study employed the same research design, methodology, and data as the primary study on PFC profiles. ^10^ In this methods section, a concise description is provided to clarify the procedures already undertaken, giving readers an understanding of the methods employed in both the primary and current studies. The text was rewritten and condensed wherever possible to avoid the issue of self-plagiarism. However, overlap could not be avoided in some instances for clarity reasons.

### Ethical considerations

The Ethical Review Board of the Open University of the Netherlands approved the study protocol (May 29th, 2019/No. U2019/03973/HVM). Both PTs and patients provided their written informed consent before participation.

### Design

In this exploratory file review study, PPTs completed a brief eligibility questionnaire covering their qualifications, registration, employment status, work experience, and workplace settings. PPTs were asked to enter anonymized data from self-selected patient files into an online survey, after obtaining written permission from the included patients. ^10^

### Participants

**PPT inclusion and recruitment:** Registered, practicing Dutch PPTs were included in this study. Two Dutch pelvic physical therapy societies published calls to participate in their newsletters to help recruit PPTs. PPTs were also recruited verbally and online through LinkedIn and Facebook. PPTs were offered a reward for participation in the form of permanent education credits.

**Patient file selection:** Dutch PPTs keep standardized patient records of patients who are self-referred or referred by a healthcare professional for pelvic physical therapy. Each PPT could include data from up to 30 patient files of pregnant, parous, and nulliparous patients aged between 18 and 45. These patients were either currently being treated or had received treatment within the past year. To explore the unbiased influence of pregnancy and parity, eligible pregnant patients were those expecting their first child; parous patients were those who had given birth no more than two years ago and were not pregnant; and nulliparous patients had no children and were not pregnant. The initial idea was to ask PPTs to include ten pregnant, ten parous, and ten nulliparous patients to explore profiles across equally large groups. However, this request was challenging due to the different caseloads and specializations. Therefore, it was decided that all submissions with up to 30 patients were deemed acceptable.

### Measurement instrument

PFCs and PFC-related distress recorded in patient files were to be entered in an online survey. Age, pregnancy status, gestational age in months, parity status, and the number of previous pregnancies were recorded for pregnant patients. The number of children, the age of the youngest child, and the number of vaginal deliveries and cesarian sections were inventoried for parous patients. A catalog was provided to assist in extracting the recorded PFCs and PFC-related distress from patient files before entering them in the online survey. PFC symptoms and definitions of the types of PFC-related distress from the conceptual model were included in the catalog (see Table 1).

**Table 1.** Reference catalog of pelvic floor symptoms and psychological distress definitions.

Catalog items		Symptoms and Definitions	NVFB Diagnoses	Categorized PFC
**Pelvic Floor Complaints (PFC)**				
**1**	**Urinary Incontinence**	Involuntary loss of urine when sneezing, coughing, laughing, jumping etc., Urge incontinence, Loss of urine during sex, Unnoticed loss of urine, or Leakage after micturition	Stress Urinary Incontinence (SUI) Urge Urinary Incontinence (UUI) Mixed Urinary Incontinence (MUI)	**Urinary Incontinence**
**2**	**Fecal Incontinence**	Involuntary loss of feces, Unnoticed loss of feces, Involuntary loss of feces when sneezing, coughing, laughing, jumping etc.	Fecal Incontinence Soiling Stamping	**Fecal Incontinence**
**3**	**Flatus**	Inability to hold wind, Excessive intestinal gas formation and bother of flatus, Flatus related to specific types of food	Anal Flatus	
**4**	**Dysfunctional Voiding**	Hesitation, Incomplete voiding, Pushing to urinate, Changed flow, Postponing micturition, Not taking time for micturition	Dysfunctional Voiding Urinary Retention	**Micturition Problems**
**5**	**Urge/Frequency (micturition)**	Frequent micturition, Frequent urge, Imperative urge, Painful urge, Interstitial Cystitis	Frequency Interstitial Cystitis Overactive Bladder	
**6**	**Urinary Tract Infections**	Burning pain during micturition, Loss of control over urge, Sense of pressure in/on bladder, Frequent urge	Urinary Tract Infections	
**7**	**Urge/Frequency (defecation)**	Empty insistence, Incomplete emptying, Frequent urge, Difficulty postponing urge	Fecal Urge Frequency	**Defecation Problems**
**8**	**Constipation**	Having to push (hard) to empty bowel, Feeling bloated or full, Abdominal pain because of full bowel, No appetite, Postponing defecation, Overflow	Constipation Slow Transit Spastic Colon	
**9**	**Anal Complaints**	Hemorrhoids, Fissures, Pain during defecation, Anal cramp	Anal Cramp Anal Fissures	
**10**	**Vaginal Pelvic Organ Prolapse**	Sense of vaginal pressure, Heaviness, Sense of ball protruding, Visible ball protruding, Tampons do not stay put, Cystocele, Rectocele, Uterus prolapse	Cystocele Descensus Uteri Prolapse Rectocele	**Pelvic Organ Prolapse**
**11**	**Rectal Pelvic Organ Prolapse**	Sense of anal pressure, Protrusion of rectum through anus	Enterocele Prolapse	
**12**	**Low Back/Pelvic Pain**	Low back pain, Hernia, Pelvic pain, SI-joint pain, Buttock pain, Groin pain, Hip problems, Pain that spreads into the legs, Starting stiffness	Low Back Pain Pelvic Pain Pregnancy-related Pelvic Pain (pre- and postnatal) ^30^ Luxation	**Pelvic Pain**
**13**	**Genital Pain**	Perineal pain, Vulvodynia, Painful scars	Pelvic Floor Pain Scar Tissue Lacerations	
**14**	**Coccyx Pain**	Coccyx pain during sitting, sitting down and standing up from sitting	Coccygodynia	
**15**	**Painful Intercourse**	Penetration pain, Inability to use tampons, Inability to insert a finger in vagina, Vaginismus, Deep vaginal pain during penetration sex, Orgasm problems resulting from pelvic floor muscle tension	Dyspareunia Vaginismus Vulvar Pain Syndrome Vulvar Vestibulitis Syndrome	**Painful Intercourse**
**Psychological Distress**				
**19**	**Loss of Control**	Being unable to control one’s feelings or actions		
**20**	**Feeling Insecure**	Feeling uncertain or anxious about oneself		
**21**	**Feeling Wronged**	Being treated unfairly, or unjustly		
**22**	**Feeling Helpless**	The inability to defend oneself or act without help		
**23**	**Sexual Distress**	Distress related to problems in the sexual response cycle that prevents someone from experiencing satisfaction from sexual activities		
**24**	**Feeling Angry**	Feeling or showing strong annoyance, displeasure, or hostility		
**25**	**Feeling Disappointed**	Feeling sad or displeased because someone or something has failed to fulfil one’s hopes and expectations		

### Procedure

The file review study was embedded in the secure web-based application O4U ( https://o4u.ou.nl/en/node/234). The survey functionality in O4U was pilot-tested and adapted by the principal investigator, then retested and approved by two other PPTs. Participating PPTs received access to an information letter explaining the study’s background and objectives via an online link on the study platform. PPTs completed a short eligibility survey after signing the online informed consent form to gain access to the patient questionnaires. Download links were provided to access the informed consent forms for prospective patients. Printed informed consent forms were sent by regular mail on request. The patients signed informed consent forms that were posted to the principal investigator (AB) and stored in compliance with applicable privacy laws and regulations. Entering data online took a few minutes per patient. After a sensitivity analysis showed no relevant differences, the test data were added to the dataset to increase the sample size. Data were collected between February 2022 and October 2023.

### Data-analysis

Data were analyzed using SPSS 28, ^33^ Excel, and R. ^34^ Before the analysis, the entered PFCs were aligned with the categorization used in previous research, as indicated in Table 1. ^26^ The sum scores of each PFC were calculated based on the recorded number of symptoms shown in the catalog. Scores of ‘0’ indicated absence in patient files, and all other scores indicated presence, indicated by ‘1’, resulting in a data set of binary variables.

Descriptive statistics were calculated in SPSS and Excel, including the recorded frequencies and percentages of PFC types and PFC-related distress in the total sample and subgroups of pregnant, parous, and nulliparous patients. To account for their dichotomous measurement level, pairwise correlations between the proportions of the outcome variables were computed using the formula: r = (y(11) – y1*y2)/(y1*(1-y1)*y2*(1-y2))^(1/2). ^25^ In this formula, y(11) represents the proportion of patients with both complaints recorded, y1 is the proportion of patients for whom complaint ‘1’ was recorded, and y2 reflects the proportion of patients for whom complaint ‘2’ was recorded.

Latent Class Analysis (LCA) was performed to explore and identify characteristic response patterns among the recorded PFCs and PFC-related distress and to cluster patients according to these patterns to identify indicative, typical, and relevant patient profiles. The recommended sample size for a (multilevel) LCA is between 300 and 1000 respondents. ^35^ In this LCA, PFC and psychological distress (PFC + PD) profiles were explored by adding recordings of the seven PFC-related distress types to those of the seven common PFCs. The LCA model selection process is explained in detail in the supplementary material (see Appendix B). Based on statistical considerations from log-likelihood indices and the Bayesian Information Criterion (BIC), the emergent models were explored to identify a preference for a single- or multilevel model (see Appendix B). The subsequent statistical model selection procedure was iterative, ^36^ aiming to identify a plausible number of classes while exploring potential dependencies among patients clustered by group and by PPT practice. This exploration was necessary to assess potential differences between PPTs in PFCs and PFC-related distress recordings, to identify PFC + PD profiles, and to determine the likelihood of encountering these profiles among pregnant, parous, and nulliparous patients. ^36^ The LCA model selection procedure was extended to incorporate theoretical considerations and to consult four expert PPTs to interpret the emergent LCA models in terms of clinical relevance.

## Results

After an extensive recruitment period to generate a sufficiently large dataset for our exploratory analyses, 22 PPTs ultimately participated in this study. On average, they reported 12.59 years of work (SD = 6.48) in private practice. Three PPTs also worked in hospitals and healthcare centers. Data on 366 patients were included in the analyses, rendering the sample relatively small for exploratory multilevel LCA. Given the specific patient populations and specialization of PPTs, the intended data stratification into three equally large groups of pregnant, parous, and nulliparous patients failed. Data from 76 pregnant patients with a mean age of 30.11 years (SD = 3.68) and gestation age of 23.59 weeks (SD = 7.64), 205 parous patients with a mean age of 32.83 years (SD = 4.11), and on average 1.61 children, and 85 nulliparous patients with a mean age of 28.69 years (SD = 8.17) were analyzed.

In the observed recording patterns, insecurity was the most frequently recorded type of PFC-related distress. Loss of control and sexual distress followed as the second and third, before disappointment, helplessness, anger, and finally feeling wronged. The highest percentage of loss of control was recorded for patients with fecal incontinence. The highest percentages of sexual distress and disappointment were recorded for patients experiencing painful intercourse. Table 2 shows the percentages of recorded types of PFC-related distress for the total sample and each specific PFC.

**Table 2.** Distribution of the types of pelvic floor complaint-related distress in the full sample and across pelvic floor complaints.

		Types of Pelvic Floor Complaint-related Distress						
Complaints	Sample size	Loss of Control	Feeling Insecure	Feeling Wronged	Feeling Helpless	Sexual Distress	Feeling Angry	Feeling Disappointed
	N	%	%	%	%	%	%	%
**Full Sample**	**366**	14.8	**27.4**	1.6	10.0	14.1	3.0	12.5
Urinary Incontinence: **UI**	**125**	20.0	**33.6**	2.4	9.6	14.4	4.0	13.6
Fecal Incontinence: **FI**	**20**	**30.0**	**35.0**	0.0	15.0	5.0	5.0	5.0
Micturition Problems: **MP**	**131**	18.3	**30.5**	0.0	13.7	18.3	3.1	15.3
Defecation Problems: **DP**	**113**	15.9	**29.2**	0.0	10.6	18.6	3.5	14.2
Pelvic Organ Prolapse: **POP**	**72**	15.3	**25.0**	1.4	6.9	9.7	2.8	11.1
Pelvic Pain: **PP**	**267**	16.1	**27.3**	2.2	12.0	13.1	3.7	13.5
Painful Intercourse: **PI**	**86**	20.9	**45.3**	0.0	16.3	**34.9**	5.8	**27.9**

Note: The types of PFC-related distress with recordings of more than 25% in the full sample and per pelvic floor complaint are highlighted in bold.

Table 3 shows the pairwise correlations between PFCs and types of PFC-related distress. Positive correlations indicate complaints likely to be (not) recorded together, and negative correlations indicate that one complaint is likely to be recorded but not the other. The correlation between painful intercourse and sexual distress was moderate. A weak to moderate correlation was found between painful intercourse and insecurity and disappointment. Weak positive and negative correlations were observed between various PFCs and PFC-related types of distress.

**Table 3.** Pairwise correlations in the full sample between pelvic floor complaints and pelvic floor complaint-related distress.

		Types of Pelvic Floor Complaint-related Distress					
Complaints	Loss of Control	Feeling Insecure	Feeling Wronged	Feeling Helpless	Sexual Distress	Feeling Angry	Feeling Disappointed
Urinary Incontinence: **UI**	**0.11**	**0.10**	**-0.04**	-0.01	**0.00**	**0.04**	**0.02**
Fecal Incontinence: **FI**	**0.10**	**0.04**	-0.03	**0.04**	-0.06	**0.03**	-0.05
Micturition Problems: **MP**	**0.10**	**0.05**	-0.10	**0.09**	**0.09**	**0.00**	**0.06**
Defecation Problems: **DP**	**0.02**	**0.02**	-0.09	**0.01**	**0.08**	**0.02**	**0.03**
Pelvic Organ Prolapse: **POP**	**0.01**	-0.03	-0.01	-0.05	-0.06	-0.01	-0.02
Pelvic Pain: **PP**	**0.06**	-0.01	**0.08**	**0.10**	-0.05	**0.07**	**0.05**
Painful Intercourse: **PI**	**0.10**	**0.22**	-0.07	**0.11**	**0.33**	**0.09**	**0.26**

[i] Note: **N = 366.** In bold, positive correlations are highlighted. In green, positive correlations above 0.10 are indicated. In red, negative correlations below 0.10 are indicated.

### Latent class analysis

The model selection procedure indicated that the data best supported a 5-class multilevel group LCA. This model grouped pregnancy and parity status and used PPT as a cluster variable (see Appendix B for more details on model selection). Model selection was based on both statistical indicators (log-likelihood, BIC) and theoretical considerations. In the theoretical considerations, the plausibility of PFC combinations was given more weight than that of PFC-related distress combinations, because the somatic PFC recordings were assumed to be more robust and structured than the PFC-related distress recordings.

Similar to the earlier work, the five indicative classes (profiles) found were named after the one or two most distinct pelvic floor complaints, with the highest likelihood. ^10^ As the somatic component of the profiles showed a strong correspondence with the earlier work, the same names and colors were used to facilitate interpretation and comparison between profiles with and without indicators of psychological burden. In Figure 1, the PFC + PD profiles are shown alongside the PFC-only profiles from our earlier work. ^10^

**Figure 1.**
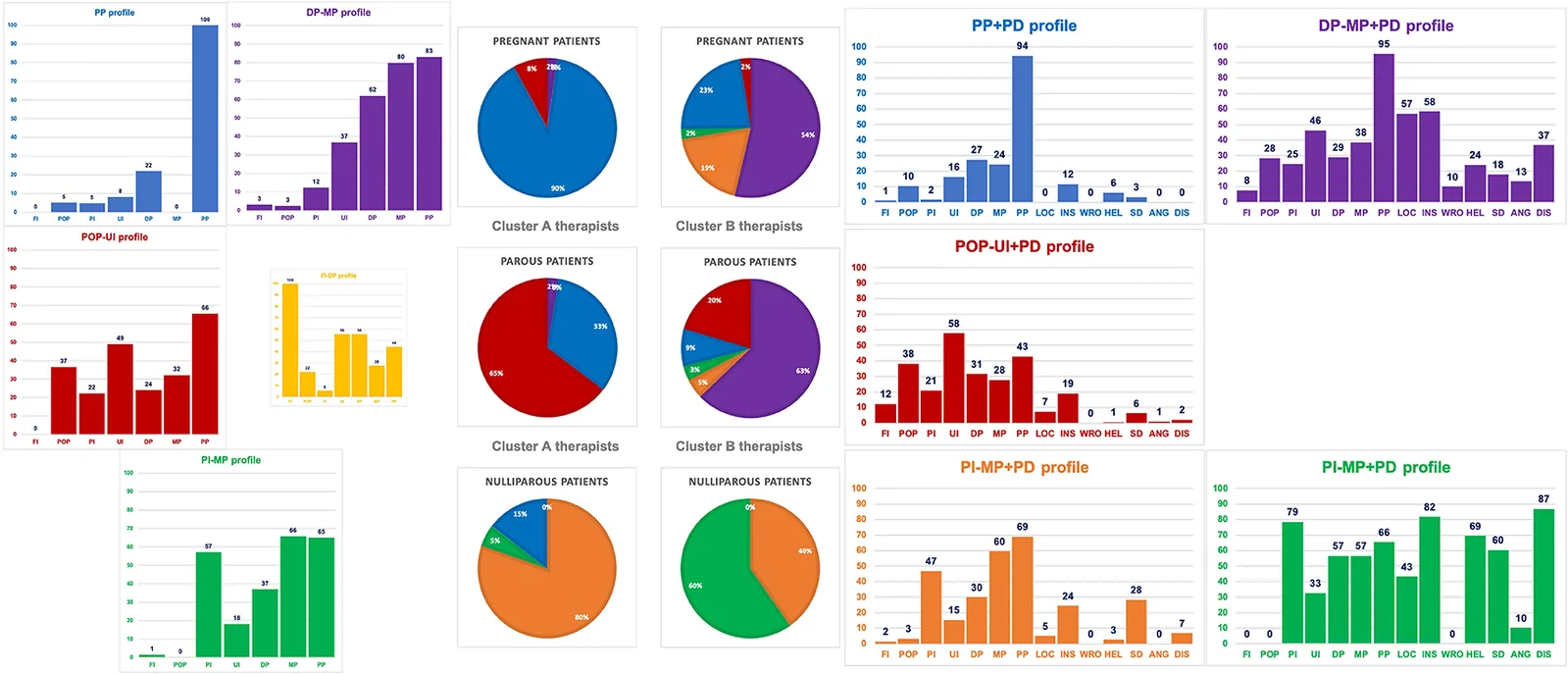
Five relevant pelvic floor complaint and pelvic floor complaint-related distress profiles, and the likelihood of each profile in each patient group, as recorded by pelvic physical therapists. Note: FI = Fecal incontinence, POP = Pelvic organ prolapse, PI = Painful intercourse, UI = Urinary incontinence, DP = Defecation problems, MP = Micturition problems, PP = Pelvic pain. PFC = Pelvic floor complaint, PD = Psychological distress. The bar charts show the likelihood, in %, that each complaint is present in a profile. On the left, the original PFC-only profiles are shown for comparison with the combined PFC + PD profiles on the right. In the middle, the likelihood of each profile’s presence in pregnant, parous, and nulliparous patient files is shown for the two groups of pelvic physical therapists. Cluster A therapists recorded very little PFC-related distress, as indicated in the pie charts on the far left, whereas Cluster B therapists recorded more PFC-related distress.

Upon inspection of our data, two clusters of therapists were unexpectedly identified, and the clustering appeared to be related to differences in behavior in the overall frequency of recorded distress among PPTs. The class probabilities for each therapist cluster are also shown in Figure 1, split by parity status. The left column of three pie charts in the center of Figure 1 shows the distribution of PFC + PD profiles across patient groups among Cluster A PPTs, who were generally found to record psychological distress less often. The right column of the three pie charts shows the distribution of PFC + PD profiles across patient groups among Cluster B PPTs, who were generally found to record psychological distress more often.

The five indicative PFC + PD profiles found were: 1) pelvic pain (blue); 2) defecation and micturition problems (purple); 3) pelvic organ prolapse and urinary incontinence (red); 4) painful intercourse and micturition problems (green); and 5) painful intercourse and micturition problems (orange). In the pelvic pain PFC + PD profile (blue), the likelihood of pelvic pain was high, with low probabilities of the other PFCs. No substantial distress, other than insecurity, was recorded in this profile. Cluster A PPTs most frequently recorded this profile for pregnant patients, and with a lower probability for parous and nulliparous patients. The defecation and micturition problems PFC + PD profile (purple) also included high probabilities of pelvic pain, with moderate likelihood of defecation and micturition problems, pelvic organ prolapse, painful intercourse, and urinary incontinence, but not fecal incontinence. All types of distress were substantially recorded with this profile, in particular loss of control, insecurity, and disappointment. Cluster B PPTs most frequently recorded this PFC + PD profile among pregnant and parous patients, suggesting that this profile may not be exclusive to pregnant patients. The pelvic organ prolapse and urinary incontinence PFC + PD profile (red) appeared most characteristic of parous patients. No substantial psychological distress was recorded with this profile apart from insecurity. Cluster A PPTs recorded this profile most frequently. Next to the painful intercourse and micturition problems PFC + PD profile (green), a similar PFC + PD profile (orange) emerged. Both these green and orange painful intercourse and micturition profiles were most frequently recorded for nulliparous patients. One painful intercourse and micturition problems PFC + PD profile (green) included high probabilities in psychological distress, particularly loss of control, insecurity, helplessness, sexual distress, and disappointment. The other painful intercourse and micturition problems PFC + PD profile (orange) included lower probabilities for various similar PFCs, and lower probabilities in psychological distress, including mainly insecurity and sexual distress. For both painful intercourse and micturition problems PFC + PD profiles, cluster A and B PPTs appeared to record similar types of distress at different frequencies, suggesting that cluster B PPTs were more likely to record distress than cluster A PPTs. The results were discussed with four PPT experts, who identified the five PFC + PD profiles as clinically relevant.

### Comparison between the PFC-only and PFC + PD profiles

The pelvic pain (blue) and defecation and micturition problems (purple) profiles remained most characteristic of pregnant patients. The two pelvic pain profiles (blue) showed high probabilities of pelvic pain and low probabilities of the other PFCs. Both defecation and micturition problem profiles (purple) included high probabilities of pelvic pain, and after adding psychological distress, the likelihood of defecation and micturition problems appeared lower. In both pelvic organ prolapse and urinary incontinence profiles (red), the probability of the various types of PFCs and their diffuse character was similar. The fecal incontinence and defecation problems profile (yellow) disappeared completely after psychological distress was added to the analyses. This could be due to the relatively small group of twenty parous patients with recorded fecal incontinence, implying a less robust profile. In contrast, cluster B therapists recorded the micturition and defecation PFC + PD profile (purple), which includes a higher probability of defecation problems, also in files from parous patients. In a new study with a larger sample, the fecal incontinence and defecation problems profile (yellow) might re-emerge. In this multilevel LCA, the fecal incontinence recordings appear to have been merged into the pelvic organ prolapse and urinary incontinence PFC + PD profile (red), confirming these PFCs as characteristic of parous patients. The defecation problem recordings may have been incorporated into the micturition and defecation problems profile (purple), which, in this study, appeared to also be frequently recorded by cluster B therapists for parous patients. The multilevel painful intercourse and micturition problems PFC + PD profile (green) indicated similar high probabilities in PFCs to the PFC-only profile (green).

### Discussion

Based on data from patient files, frequencies and associations between PFCs and PFC-related types of distress were explored to generate hypotheses about relevant PFC + PD profiles. Five indicative PFC + PD profiles related to pregnancy and parity were found. Based on observed recording patterns and limited documentation of PFC-related distress in patient files, the results need to be interpreted with caution. Two PFC + PD profiles appeared most characteristic of pregnant patients, one of parous patients, and two of nulliparous patients. While some attention is given to PFC-related distress, it seems to remain underrepresented in the patient files. In the profiles, sparse PFC-related distress recordings could appear to be associated with observed PPT recording behavior and the nature, number, and combinations of probable PFCs.

PPTs appeared mostly to record three types of PFC-related distress from the conceptual model developed in previous qualitative research: ‘feeling insecure’, ‘loss of control’, and ‘sexual distress’ with different PFC profiles. ^27^ The other kinds of PFC-related distress from the model -‘feeling helpless’, ‘feeling wronged’, ‘feeling angry’, and ‘feeling disappointed’- were less frequently recorded. This observation raises questions about the transparency and accuracy of PPTs’ communication with their patients regarding the interpretation and documentation of expressed PFC-related distress. ^18^

The high observed frequency with which insecurity was recorded in comparison to other types of distress suggests that PPTs might easily label any distress expressed by patients as insecurity. Although it may seem positive that insecurity is recorded, it remains uncertain whether it captures the full range of distress experienced by patients. The knowledge that insecurity is related to other forms of distress ^27^ warrants further exploration of the factors contributing to the observed insecurity, as illustrated in the following examples. Losing control over one’s body may trigger a sense of insecurity due to the inability to perform daily activities. Sexual function problems may trigger insecurity for not living up to one’s own and one’s partner’s expectations during sexual interactions. Anger may trigger insecurity, leading to doubts about one’s ability to recover. Helplessness may trigger insecurity due to the loss of independence and autonomy. A sense of being wronged may trigger insecurity, fueled by the belief that the odds are against you. Disappointment may trigger insecurity due to unfulfilled hopes and expectations. ^26^ This observation suggests that various types of distress might be interpreted as a sense of insecurity, overlooking the true character and meaning of the distress experienced. Further exploration of the types of distress that appear to be related to insecurity may enhance understanding of patients’ guiding questions and contribute to the development of more tailored treatment plans.

Loss of control might be more easily recognized because this type of distress has both physical and psychological characteristics. The physical inability to perform certain physical activities often reflects a loss of control over muscle function, resulting in restrictions or the inability to engage in daily, social, and sexual activities, which can lead to distress from losing control over one’s life. Given PPT’s inventory of functional restrictions, loss of control might be acknowledged sooner than other types of PFC-related distress because of this physical aspect. Therefore, it remains uncertain to what extent PPTs acknowledge the psychological side of this type of distress. This observation suggests that PPTs might focus more on the physical than the psychological aspects of loss of control.

Based on the observed results, sexual distress might be more easily recognized in the specific and sensitive context of sexual function problems, which might explain the substantially higher frequency with which sexual distress appears to be recorded. The sensitivity of discussing sexual function problems might contribute to heightened attention to distress, given that it is often distressing to discuss the topic of sexual function for both patients and healthcare providers. ^37^ In addition to this observation, the dyadic nature of sexual function problems in the context of relationships, including partner involvement and behavior, and the wish for children, might also highlight this type of distress from a social perspective. This observation suggests that attention to Sexual distress might be more easily recognized in the specific and sensitive context of sexual function problems, but might be overlooked in the context of other PFCs.

No literature was found on research that unravels PFC + PD profiles; therefore, it is not possible to compare the results with previous findings. However, the preliminary results in this study suggest the potential of distinct sets of PFC-related distress types for each profile. The results suggest some attention to PFC-related distress in pelvic physical therapy practice in line with the biopsychosocial perspective. More distress appeared recorded in two: the defecation and micturition problems (purple) and the painful intercourse and micturition problems (green) PFC + PD profiles with a higher probability of multiple PFCs than in the other: the pelvic pain (blue), the pelvic organ prolapse and urinary incontinence (red), and the painful intercourse and micturition problems (orange) PFC + PD profiles. A higher likelihood of multiple PFCs being presented together might automatically trigger a greater alertness to psychological distress, which may render the recording of distress more likely. In the blue, red, and orange profiles, one for each pregnancy and parity group, PFC-related distress appeared sparsely recorded, despite the high likelihood of one or more distinct PFC types. These findings do not appear to fully reflect the different kinds of PFC-related distress of pregnant, parous, and nulliparous patients, and the higher levels of PFC-related distress associated with, and predictive of, receiving pelvic physical therapy treatment as identified in prior research. ^11^^,^^26^^,^^27^ Women receiving pelvic physical therapy treatment, particularly parous patients, expressed high levels of anger and feeling wronged, which did not appear in the pelvic organ prolapse and urinary incontinence PFC + PD profile (red). Nulliparous patients expressed high levels of helplessness, which seems only recorded in the painful intercourse and micturition problems PFC + PD profile (green). Pregnant patients expressed high levels of loss of control, which appeared only recorded in the defecation and micturition problems PFC + PD profile (purple). ^26^ The low likelihood of PFC-related distress recorded in parous patients’ profiles, compared to the other profiles, raised the question of whether the distress experienced by parous patients may not have been evident enough or perhaps even taken for granted and therefore not recorded. This stands in high contrast with the recent evidence of distinct and overwhelming types of distress being experienced by parous patients who receive pelvic physical therapy treatment. ^26^^,^^27^ A possible explanation might be that the treatment of parous patients primarily focuses on recovering pelvic floor muscle function after varying degrees of pelvic floor damage, including stretching, lacerations, and episiotomies, which might prompt more somatically oriented guiding questions and treatment approaches. This observation seems to warrant further exploration of the reasons and opportunities for recording or not recording psychological distress in pelvic physical therapy practice. The findings in previous research and the observed PFC + PD profiles suggest the existence of characteristic PFC + PD profiles for specific patient groups.

When PFC-related distress was not recorded, this did not necessarily imply its absence, as patients may not have expressed, discussed, or acknowledged it with PPTs, and it may not have been recorded for various reasons. The observed overall sparseness of PFC-related distress recordings may be due to various factors, such as a lack of recording space for psychological issues in patient files, or it may imply limited attention to psychological problems and PFC-related distress in pelvic physical therapy education. Arguments for or against recording psychological distress in patient files often revolve around the balance between legal and ethical obligations, quality of care, client rights, and privacy. ^1^^,^^38^ Not recording distress may be related to the subject’s sensitivity, a lack of patients’ permission to record their distress out of fear of stigma and taboo, or fear of breaches of privacy and confidentiality that accompany discussing these topics. Finally, the PPT’s subjective judgment regarding the relevance of distress to treatment and prognosis may be decisive when recording psychological distress in patient files. Therefore, the profiles provide only a first tentative, hypothetical impression of the types of PFC-related distress associated with each profile, which need to be investigated in larger samples to assess their robustness. It is also uncertain whether the conspicuous absence of psychological distress in the definition of pelvic floor dysfunction in comparison to the inclusion of distress in the definition of sexual dysfunction relates to the low recordings of PFC-related distress in patient files. ^9^^,^^39^ The sparse observed PFC-related distress recordings and expressed concerns regarding inadequate communication of patients’ pain and behavioral responses may indicate a necessity to re-evaluate the application of the biopsychosocial framework within pelvic physical therapy education and practice. ^10^^,^^12^ It may be necessary to educate PPTs on how to better recognize various types of psychological issues and PFC-related distress and how to communicate them. Further research is needed to examine the influence of the current definition, communication, and education on PPTs’ recording behavior of psychological distress. In future research, patient reports should be included in analyses to verify the accuracy of PPT-recorded PFC-related distress. In addition to the presence of PFC-related distress, the severity, duration, and impact of distress should be considered to enhance the clinical utility of the combined profiles and improve the biopsychosocial treatment approach in pelvic physical therapy practice. These observations and contemplations suggest that when more attention is paid to psychological distress in PPT education, documentation, and the definition of pelvic floor dysfunction, the recording of psychological distress in patient files might increase.

### Strengths and limitations

A file review design was chosen because it provides insight into and enhances understanding of healthcare processes, including adherence to guidelines and protocols, the completeness and accuracy of clinical documentation, and opportunities for feedback and education improvement. In the exploratory phase of research, a file review study is a practical and efficient approach. File review studies are designed to assess and improve the quality of care. ^40^ For this reason, in this study, the attention to psychological distress in pelvic therapy practice was explored, given the intended biopsychosocial treatment approach, and our interest in the documentation of psychological distress in patient files. A file review study respects the privacy and anonymity of patients, including those who may not have initially wanted to participate, and avoids recall bias. File review studies are conducted to identify patterns in patient data, informing critical healthcare decisions, such as patient treatment outcomes and multidisciplinary collaboration. File review studies are valuable in situations where clinical details relevant to treatments may be easily overlooked or deemed irrelevant, and when the goal is to gain an overview of treatment content. Given the exploratory and hypothesis-generating nature of this research, a retrospective study design was considered the most appropriate at this stage. Data were collected in alignment with the study’s aim.

An important methodological limitation of this research is the relatively small sample size. In multilevel LCA, a substantially larger sample size (more toward 1000) is preferred to increase model stability and reduce the risk of overfitting. ^35^ Given the exploratory, hypotheses-generating, and innovative character of this study and the experienced recruitment challenges, the relatively small sample size was deemed acceptable for generating a first indication of the existence and content of PFC + PD profiles. The results should thus be cautiously interpreted and treated as indicative and hypothetical. Therefore, this research should be repeated with a larger sample of PPTs and patients to test whether the identified profiles are robust, statistically significant, and clinically relevant. In addition, insight into the observed psychological distress-recording behavior of PPTs is needed to assess whether this behavior is representative of clinical reality and, if not, why not. Comparing recorded complaints with data collected from patients whose files are included in the research would help verify the recorded data against lived experiences. Subsequently, the accuracy of PPTs’ biopsychosocial approaches could be improved.

A second limitation of this research is that the results may not generalize due to the stratified, non-representative sample and potential selection bias, as PPTs self-selected the included files. In addition, PFC-related distress was sparsely recorded and may have been incompletely, inconsistently, under-, or not concisely documented for unknown reasons, despite the given instructions, potentially resulting in information bias. In addition to selection and information bias, inter-rater variability in documentation may have influenced the results. The identified PFC + PD profiles are based on PPTs’ documentation of psychological distress in patient files, which may not be indicative of clinical reality but more of documentation behavior. Including patient-reported PFC-related distress in follow-up research may help to interpret recorded PFC-related distress with greater certainty. In addition, the number of participating PPTs in this study was small. The selection of PPTs may not be representative of the Dutch PPT population, and their patient selection may not reflect the types of patients treated in general PPT practices. These factors may limit generalizability across the Netherlands. Therefore, more research is needed to address potential issues of generalizability.

## Conclusion

This exploratory, hypothesis-generating retrospective file review study provides initial insight into PPTs’ recording behavior and patterns of PFC-related distress alongside PFCs in patient files. While some PFC-related distress was recorded, especially insecurity, loss of control, and sexual distress, the overall observed recording was limited, and PFC-related distress was potentially under-recorded. Results suggest that attention to predisposing psychological issues related to PFCs and PFC-related distress in education and documentation might further enhance PPTs’ biopsychosocial approach in history-taking, (multidisciplinary) treatment selection and approaches, and facilitate collaboration with psychologists or sexologists, and enhance patient care. In forthcoming research, to confirm the generated hypotheses, further exploration of PFC + PD profiles in a larger sample, from both patient and PPT perspectives, is recommended to distinguish observed recording behavior from distinct clinical phenomena. Adding information about severity, duration, and functional restrictions may further increase the clinical utility of the profiles. PFC + PD profiles could enhance pelvic health care, benefiting both healthcare providers and patients.

## Data availability

### Underlying data

OSF: Pelvic Health Problems in Young Adult Women,

Doi: https://doi.org/10.17605/OSF.IO/ZG2BN ^41^

This project contains the following underlying data:

• Data en Codeboek PPT studie-DEF-DEF.xlsx
• Function_bootstrap.R
• Functions_plot.R
• LCA.R

Data are available under the terms of the Creative Commons Zero “No rights reserved” data waiver (CC0 1.0 Public domain dedication) ( http://creativecommons.org/publicdomain/zero/1.0/).

### Extended data

OSF: Pelvic Health Problems in Young Adult Women, doi.

https://doi.org/10.17605/OSF.IO/DWNYH ^42^

This project contains the following data:

• Appendix A
• Appendix B.pdf
• Survey PPT Study.docx
• Data en Codeboek PPT studie-DEF-DEF.xlsx
• Data en Codeboek PPT studie-DEF-DEF NL&EN.xlsx
• Function_bootstrap.R
• Functions_plot.R
• LCA.R

Data are available under the terms of the Creative Commons Zero “No rights reserved” data waiver (CC0 1.0 Public domain dedication) ( http://creativecommons.org/publicdomain/zero/1.0/).
