# Supplementary material for: Pelvic floor complaint-related psychological distress recorded by pelvic physical therapists in the Netherlands: Additional analysis of data from an exploratory file review study
Source: Open Res Eur. 2026 Oct 10;6:16. Originally published 2026 Jan 9. [Version 3] doi: 10.12688/openreseurope.22137.3 (PMC13273244; doi:10.12688/openreseurope.22137.3)
Supplement: Peer review history

# Reviewer report: Reviewer response for version 2

**DOI:** 10.21956/openreseurope.26124.r75621

**Published:** 2026-06-16

**Authors:** gianfranco lamberti

**Affiliations:** 1 University of Parma Department of Medicine and Surgery (Ringgold ID: 478519), Parma, Emilia-Romagna, Italy

The authors have satisfactorily addressed the major methodological and interpretive concerns raised in the previous review round.

 The revisions have strengthened the manuscript by deepening the discussion of study limitations, clarifying the distinction between observed documentation patterns and inferred clinical phenomena, and promoting a more appropriately exploratory, hypothesis-generating interpretation of the identified profiles. The additional discussion of recording behavior, information bias, and the potential impact of therapist-related documentation practices further enhance the manuscript's transparency and scientific rigor.

 I appreciate the authors’ efforts to acknowledge the limitations inherent in retrospective file review data and to align the interpretation of the findings more closely with the study’s exploratory nature.

 I have no further major comments. As a minor suggestion, I would encourage the authors to maintain cautious wording when referring to the identified profiles, emphasizing that they represent exploratory, documentation-based latent profiles that require prospective validation before being considered established clinical phenotypes.

 Overall, I believe the manuscript has been substantially improved and now provides a valuable exploratory contribution to the field.

If applicable, is the statistical analysis and its interpretation appropriate?

Partly

Is the study design appropriate and is the work technically sound?

Partly

Is the work clearly and accurately presented and does it engage with the current literature?

Partly

Are the conclusions drawn adequately supported by the results?

Partly

Are sufficient details of methods and analysis provided to allow replication by others?

Yes

Are all the source data and materials underlying the results available?

Yes

Reviewer Expertise:

Physical and Rehabilitation Medicine; pelvic floor and urogynecological rehabilitation; neuro-urology and neurogastroenterology; chronic pelvic pain and functional pelvic disorders

I confirm that I have read this submission and believe that I have an appropriate level of expertise to confirm that it is of an acceptable scientific standard.

# Reviewer report: Reviewer response for version 2

**DOI:** 10.21956/openreseurope.26124.r75622

**Published:** 2026-06-11

**Authors:** Phil Reed

**Affiliations:** 1 Swansea University, Swansea, Wales, UK

The authors have been very responsive to my concerns, and have acknowledged the limitations of the study very clearly.

If applicable, is the statistical analysis and its interpretation appropriate?

Yes

Is the study design appropriate and is the work technically sound?

Partly

Is the work clearly and accurately presented and does it engage with the current literature?

Partly

Are the conclusions drawn adequately supported by the results?

Partly

Are sufficient details of methods and analysis provided to allow replication by others?

Yes

Are all the source data and materials underlying the results available?

Yes

Reviewer Expertise:

Psychology; Women's Health

I confirm that I have read this submission and believe that I have an appropriate level of expertise to confirm that it is of an acceptable scientific standard.

# Reviewer report: Reviewer response for version 1

**DOI:** 10.21956/openreseurope.23962.r71209

**Published:** 2026-04-07

**Authors:** gianfranco lamberti, Levis John Kenneth Brian, Gianluca Ciardi

**Affiliations:** 1 University of Parma Department of Medicine and Surgery (Ringgold ID: 478519), Parma, Emilia-Romagna, Italy; 2 Department of Medicine and Surgery, University of Parma (Ringgold ID: 478519), Parma, Emilia-Romagna, Italy; 3 Dipartimento di Medicina e Chirurgia, Universita degli Studi di Parma (Ringgold ID: 478519), Parma, Emilia-Romagna, Italy

Reviewer Report

 This retrospective chart review investigates how pelvic physical therapists (PPTs) document pelvic floor complaints (PFCs) and associated psychological distress (PD), building on previous work on PFC-only profiles through multilevel Latent Class Analysis (LCA). The study identifies five combined PFC+PD profiles that vary based on pregnancy and parity status. The findings reveal that, although psychological distress—particularly feelings of insecurity, loss of control, and sexual distress—is present, documentation of these issues in clinical records remains limited and inconsistent. The topic is timely and clinically relevant, especially within a biopsychosocial framework of pelvic health. The manuscript is generally well organized, clearly written, and methodologically transparent. The integration of both physical and psychological aspects is an important and commendable effort.

 Main comments:

 1. Study design and robustness (Partly)

 The exploratory file review design suits the research goal. However, several limitations impact the robustness of the results.

 The sample size (N = 366) is somewhat small for a multilevel LCA that involves multiple latent classes, subgroup stratification (pregnant, parous, nulliparous), and therapist-level clustering. This raises concerns about model stability and the risk of overfitting.

 Additionally, both therapists and patient files were self-selected, which may introduce selection bias and limit the generalizability of the findings. Importantly, the key variable—psychological distress—is sparsely and inconsistently recorded, directly affecting the reliability of the analysis.

 Suggestions:

- Expand the discussion on model stability and potential overfitting
- Explicitly address selection and information bias
-  Clarify the interpretation of therapist clustering (recording behavior vs clinical reality)

2. Statistical analysis and interpretation (Partly)

 The statistical approach is suitable in principle and well explained. The use of LCA, along with model selection criteria such as BIC and log-likelihood, is methodologically solid. However, the quality of input data—especially psychological distress variables—is limited, which restricts the validity of the resulting profiles. Additionally, the identified profiles may partly reflect documentation practices rather than actual clinical entities.

 Suggestions:

- Maintain a clearly exploratory and hypothesis-generating framing
- Add a brief reflection on the measurement limitations of distress variables

3. Conclusions and clinical implications (Partly)

 The conclusions largely align with the results, especially concerning the underrepresentation of psychological distress in clinical records. However, some implications regarding:

- impact on treatment planning
- clinical applicability of the profiles
- role in biopsychosocial clinical reasoning

appear somewhat overextended, given the exploratory design and data limitations. In particular, the apparent lack of distress in some profiles may result from under documentation rather than true absence.

 Suggestions:

 Clearly distinguish between:

- observed recording patterns and inferred clinical phenomena

4. Engagement with current literature (Partly)

 The manuscript engages with relevant literature, especially regarding the biopsychosocial model and pelvic floor dysfunction. However, the introduction could better reflect the current evidence on psychological distress as a key factor influencing outcomes and possibly serving as a causal or perpetuating agent.

 Suggestions:

 Expand the introduction to include the bidirectional relationship between PFCs and psychological distress.

 Minor comments

 Consider briefly addressing inter-rater variability in documentation

 Strengths

- Clinically important and timely topic
- Clear structure and transparent methodology
- Appropriate use of multilevel modelling
- Innovative integration of somatic and psychological dimensions
- Strong open science approach (data and code availability)

Conclusion

 Overall, this is a well-conducted exploratory study with meaningful clinical relevance.

 To strengthen the manuscript and ensure scientific robustness, the following points should be addressed:

- Clarify sample size adequacy and model stability
- Acknowledge the impact of recording bias on the identified profiles
- Refine interpretation

With these revisions, the manuscript would provide a more robust, transparent, and valuable contribution to the field.

If applicable, is the statistical analysis and its interpretation appropriate?

Partly

Is the study design appropriate and is the work technically sound?

Partly

Is the work clearly and accurately presented and does it engage with the current literature?

Partly

Are the conclusions drawn adequately supported by the results?

Partly

Are sufficient details of methods and analysis provided to allow replication by others?

Yes

Are all the source data and materials underlying the results available?

Yes

Reviewer Expertise:

Physical and Rehabilitation Medicine; pelvic floor and urogynecological rehabilitation; neuro-urology and neurogastroenterology; chronic pelvic pain and functional pelvic disorders

We confirm that we have read this submission and believe that we have an appropriate level of expertise to confirm that it is of an acceptable scientific standard, however we have significant reservations, as outlined above.

# Response

**Published:** 2026-05-26

**Authors:** Alma Brand

**Affiliations:** Psychology, Open Universiteit, Heerlen, Limburg, The Netherlands

Thank you for your compliments, feedback, and helpful suggestions.

**1. Study design and profile robustness:** We have addressed your first concern about study design and profile robustness by adding an extra paragraph under the heading strengths and limitations (second paragraph) that reflects on model stability and potential overfitting. We specifically addressed selection and information bias more extensively and clarified the interpretation of therapist clustering in the third paragraph under the same heading.

**2. Statistical analysis and interpretation:** Your second concern about statistical analysis and interpretation was addressed by emphasizing our exploratory, hypothesis-generating framing more frequently throughout the paper. We have explicitly highlighted the following hypotheses, which you can find at the end of the paragraphs in the discussion:

1. Various types of distress might be interpreted as a sense of insecurity, overlooking their true character and the meaning of the distress experienced.
2. PPTs might focus more on the physical than on the psychological aspects of loss of control.
3. Sexual distress might be more easily recognized in the specific and sensitive context of sexual function problems, but might be overlooked in the context of other PFCs.
4. Characteristic PFC+PD profiles might exist for specific patient groups.
5. More attention to psychological distress in PPT education, documentation, and the definition of pelvic floor dysfunction might increase the recording of psychological distress in patient files.

We added a reflection on the measurement limitations of the distress variables, given selection and information bias, and PPT’s recording behavior in the limitations section in the second limitation paragraph.

**3. Conclusion and clinical implications:** The third concern has been addressed by clarifying the distinction between observed recording patterns and inferred clinical phenomena. The fact that we examine observed recording patterns has been more clearly emphasized throughout the paper. The conclusion and clinical implications have been adapted according to your suggestions and concerns.

**4. Engagement with current literature:** The fourth concern has been addressed by more explicitly including the bidirectional relationship between PFCs and related psychological distress in the first paragraph of the introduction. We added the sentence: “This is especially important given the bidirectional relationship between PFCs and psychological distress, and the potentially negative influence of psychological distress on treatment results”.

**Minor comments:** The inter-rater variability has been addressed in the limitations section.  We hope our revisions are satisfactory.

# Reviewer report: Reviewer response for version 1

**DOI:** 10.21956/openreseurope.23962.r71212

**Published:** 2026-03-26

**Authors:** Phil Reed

**Affiliations:** 1 Swansea University, Swansea, Wales, UK

**Review of: “ *Pelvic floor complaint-related psychological distress recorded by pelvic physical therapists in the Netherlands: Additional analysis of data from an exploratory file review study*”**

 This study reports the results of a retrospective analysis of the types of data collected by pelvic-floor physiotherapists in the Netherlands.  The results indicate that there are clusters of somatic symptoms that map onto patient types (pregnant, parous, and nulliparous), and are highly similar those found in a previous analysis.  The current study expands this analysis to include psychological distress as well as the traditional physical symptoms.  There were some relationships between aspects psychological distress and clusters of physical symptoms. 

 The motivation behind this study is very timely, as there is increasing evidence relating to the importance of psychological distress in pelvic-floor complaints.  The study report is well-written and structured, and the analytic strategy is justified in theory.  Nevertheless, there are several issues with the current version of the report that suggest that it is a little too premature. 

 A major problems is that the numbers of participants (patients) included are on the low side of the range of appropriate numbers needed for what is a very complex statistical analysis.  This potential underpowering mean that any conclusions drawn from these data must be treated with a great deal of caution.  This concern is amplified when the analysis reduces to different ‘types’ of physiotherapist (those who do, and those who do not, record psychological symptoms). As a consequence, I would like to see greater numbers of participants included in an expanded study.

 A second concern reflects the representativeness of the sample – both in terms of the physiotherapists, and also in terms of the patients’ records that they returned.  I would like to have seen some data on the degree to which these physiotherapists reflect the range of physiotherapists in the Netherlands, and, indeed, across other counties as well.  This is important if generalisations are to be made from these data.

 Equally important is to assess the range of patient records that were selected to be analysed by the various physiotherapists.  It is unclear if these records are representative of either: (1) the types of records typically kept by physiotherapists; and (2) the types of patients on whom these records are kept.  As above, without this it is unclear whether these results can be generalised.

 A more minor point is that the introduction does not really reflect the degree to which psychological distress has been recognised as a part of the cluster of issues surrounding pelvic-floor complaints.  Neither does it capture the degree to which these psychological issues may hinder treatment, and treatment outcomes.  Moreover, there is also evidence that the psychological distress can precede the development of the pelvic floor complaints.

 In summary, this is a well-motivated, and potentially important piece of work.  However greater numbers of participants are needed, and some further analysis should be conducted.   

If applicable, is the statistical analysis and its interpretation appropriate?

Yes

Is the study design appropriate and is the work technically sound?

Partly

Is the work clearly and accurately presented and does it engage with the current literature?

Partly

Are the conclusions drawn adequately supported by the results?

Partly

Are sufficient details of methods and analysis provided to allow replication by others?

Yes

Are all the source data and materials underlying the results available?

Yes

Reviewer Expertise:

Psychology; Women's Health

I confirm that I have read this submission and believe that I have an appropriate level of expertise to state that I do not consider it to be of an acceptable scientific standard, for reasons outlined above.

# Response

**Published:** 2026-05-26

**Authors:** Alma Brand

**Affiliations:** Psychology, Open Universiteit, Heerlen, Limburg, The Netherlands

Thank you for your compliments, feedback, and for sharing your concerns.

 We have addressed these as follows.

**Major concern about the sample size: **We agree with your comment. The sample size is relatively small for a multilevel LCA. It proved challenging to motivate sufficient pelvic physical therapists to participate and provide data for a large enough sample. We have addressed this issue more explicitly in the results section and indicated the data collection period in the method section, under the heading “procedure”. Given that our study’s aim was exploratory and hypothesis-generating, we decided to work with and accept the amount of data collected. We more clearly emphasized that results should be interpreted with caution and refined the phrasing regarding the unexpected emergence of different types of PPTs, which is most likely related to their recording behavior. We added a reflective paragraph on the implications of the sample size in the discussion/limitations section.

**Representativeness of the sample:** We have addressed your comment about the ‘types’ of physical therapists, noting that this distinction was based on observed behavior rather than therapist characteristics. We have adapted the phrasing in the results and discussion section, accordingly, as indicated above and addressed in the first paragraph of the discussion. Thank you for pointing this out.

**Range of patient records:** All Dutch, registered pelvic physical therapists use standardized patient records tailored to patients with pelvic floor complaints. The types of patients treated across practices vary with therapists' caseloads and specializations. All patients were referred or self-referred to a pelvic physical therapy practice. We clarified this under the heading “patient file selection”.

**Generalizability:** We have further elaborated on generalizability for therapists and the selected patient files in the last paragraph of our limitations section.

**Relationship between PFCs and psychological distress:** Your comment on the relationship between PFCs and psychological distress has been addressed more explicitly in the first paragraph of the introduction by adding a sentence: “This is especially important given the bidirectional relationship between PFCs and psychological distress, and the potentially negative influence of psychological distress on treatment results”. Thank you for pointing this out.

 In general, we have adapted the tone of the results and discussion to be more tentative than conclusive, emphasizing the exploratory, hypothesis-generating character of the study and further highlighting the limitations related to sample size, representativeness, and generalizability. Follow-up research with a larger sample size is definitely needed to examine the validity and robustness of the results.
